# Supplementary material for: DNA polymerase stalling at structured DNA constrains the expansion of short tandem repeats
Source: Genome Biol. 2020 Aug 21;21:209. doi: 10.1186/s13059-020-02124-x (PMC7441554; doi:10.1186/s13059-020-02124-x)
Supplement: Supplementary file 1 — Additional file 1: Supplementary Information comprising: Figure S1. Primer extension assay quality controls. Figure S2. The kinetics of DNA synthesis highlights structure-dependent transient and persistent stalling events. Figure S3. Supervised machine learning approach to structure prediction from DNA polymerase stalling events. Figure S4. Inferring STR structures from DNA polymerase stalling events. Figure S5. Validation of predicted STR structures. Figure S6. The DNA polymerase remodels STRs during DNA synthesis. Figure S7. Identification of sequence variants within the pool of newly synthesised DNA molecules. Figure S8. STR structures impact the frequency and nature of expansion/contraction events. Figure S9. STR structures impact the frequency and nature of nucleotide substitution events. Figure S10. Structure-dependent nucleotide substitution preferences. Figure S11. DNA polymerase stalling at DNA structures predicts abundance and length of STRs in the human genome. Figure S12. DNA polymerase stalling at DNA structures predicts abundance and length of STRs in eukaryotic genomes. Figure S13. DNA polymerase stalling at DNA structures predicts STR stability in the human genome. Figure S14. Structured STRs are prone to sequence variation in the human genome. Table S1. Oligonucleotides used for the validation of STR structures by 1H NMR spectroscopy. [file 13059_2020_2124_MOESM1_ESM.pdf]

ADDITIONAL FILE 1

Supplementary information

for

DNA polymerase stalling at structured DNA constrains the expansion of Short Tandem Repeats

Pierre Murat[1,*], Guillaume Guilbaud[1] and Julian E. Sale[1,*]

[1] MRC Laboratory of Molecular Biology, Francis Crick Avenue, Cambridge, CB2 0QH, UK

[*] to whom correspondence should be addressed: pmurat@mrc-lmb.cam.ac.uk or jes@mrc-lmb.cam.ac.uk

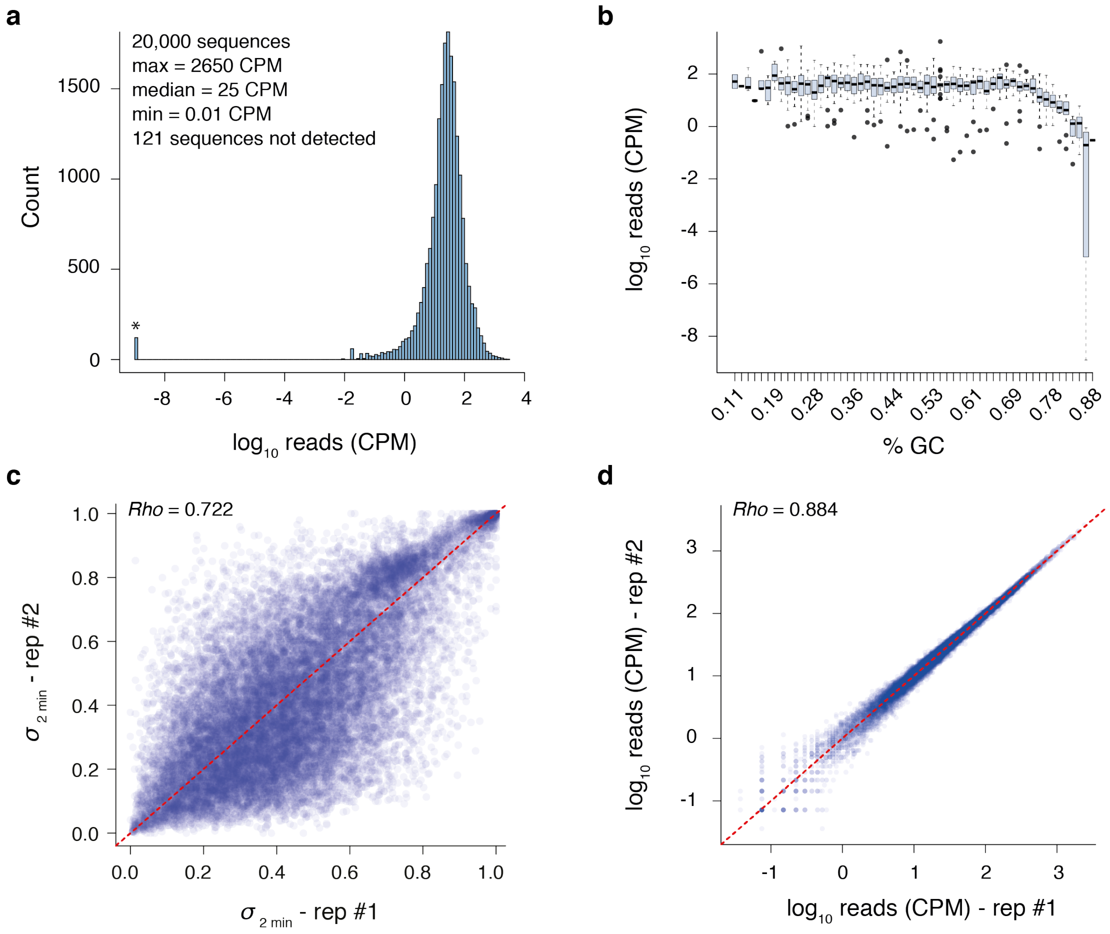

Figure S1. Primer extension assay quality controls. (a) Distribution of averaged read counts, in counts per million (CPM), associated with the 20,000 sequences from the DNA library obtained from the sequencing of the parental library, *i.e.* single-stranded DNA templates before T7 DNA polymerase extension. 121 sequences (0.6% of the library) not detected in the parental library were detected after enrichment in the stalled fractions. These sequences are marked * to indicate the assignment of an arbitrary value of one over the total number of reads. **(b)** Impact of GC richness on sequence representation for the 1,000 negative control sequences, *i.e.* random sequences of varying GC content, showing that extreme GC contents affect representation only moderately. **(c)** Reproducibility of the sequencing and stall score computing pipeline. Example of correlation for stall scores obtained in between duplicates (stall scores at 2 min for all 20,000 sequences). It is noteworthy that the variability observed in the stall scores between duplicates reflects the variation in the ability of a sequence to stall the polymerase rather than technical limitations associated with the library preparation steps. Indeed, a strong correlation **(d)** between the sequencing representations of the 20,000 sequences within the parental library is observed. Reported correlations (*Rho*) are Spearman correlation coefficients.

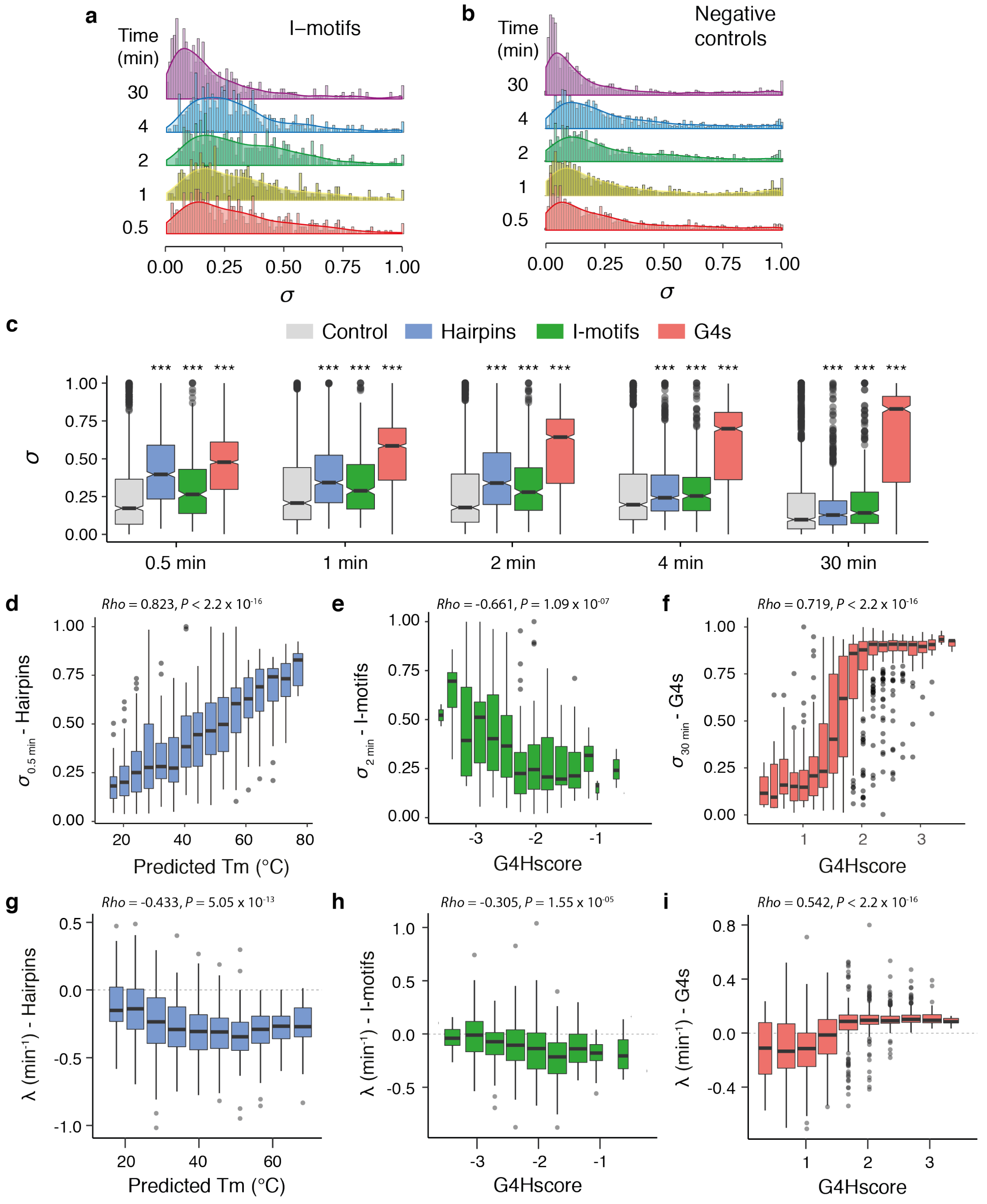

Figure S2. The kinetics of DNA synthesis highlights structure-dependent transient and persistent stalling events. Distribution and density plots of the computed stall scores associated with the i-motifs **(a)** and random control sequences **(b)** at different time points. **(c)** Stall scores associated with designed structured sequences (Hairpins in blue, i-motifs in green and G4s in red) are globally higher than scores associated with the negative control sequences (in grey), *i.e.* the set of random sequences of varying GC content, at each time point, indicating structure- rather than sequence-dependent stalling. Correlations between predicted stabilities of hairpins **(d)**, i-motifs **(e)** and G4s **(f)** and stall scores. Predicted melting temperatures (*Tm*) for hairpins were computed using the formula $Tm = L \times (2\times\%GC + 2)$, where *L* is the length of the stem of the hairpin and %GC

the GC content of the sequence (Marmur and Doty, 1962). For tetrahelical structures we computed a G4Hscore which is a quantitative estimation of G-richness and G-skewness that correlate with the folding propensity (Bedrat et al., 2016). Briefly, each position in a sequence is given a score between -4 and 4. To account for G-richness, a single G is given a score of 1, in a GG sequence each G is given a score of 2; in a GGG sequence each G is given a score of 3; and in a sequence of 4 or more Gs each G is given a score of 4. To account for G-skewness, Cs are scored similarly but values are negative. While high positive G4Hscore indicate G4 formation, low negative values indicate i-motifs formation. We found that the correlations between stall scores and structure thermodynamic stabilities are best described for hairpins, i-motifs and G4s at 0.5, 2 and 30 min respectively. Correlation between predicted stabilities of hairpins (**g**), i-motifs (**h**) and G4s (**i**) and the constants associated with the kinetic of DNA synthesis at these structures. Kinetic constants were extracted by fitting the variation of stall scores overtime using exponential growth/decay functions: $\sigma(t) = \sigma_0 . e^{\lambda t}$. Centre lines denote medians, boxes span the interquartile range, and whiskers extend beyond the box limits by 1.5 times the interquartile range. *P* values for the comparison of the distributions were calculated using the Kolmogorov–Smirnov test, ***$P <$ 0.001. *Rho* correlations reported in panels (**d-i**) are Pearson correlation coefficients.

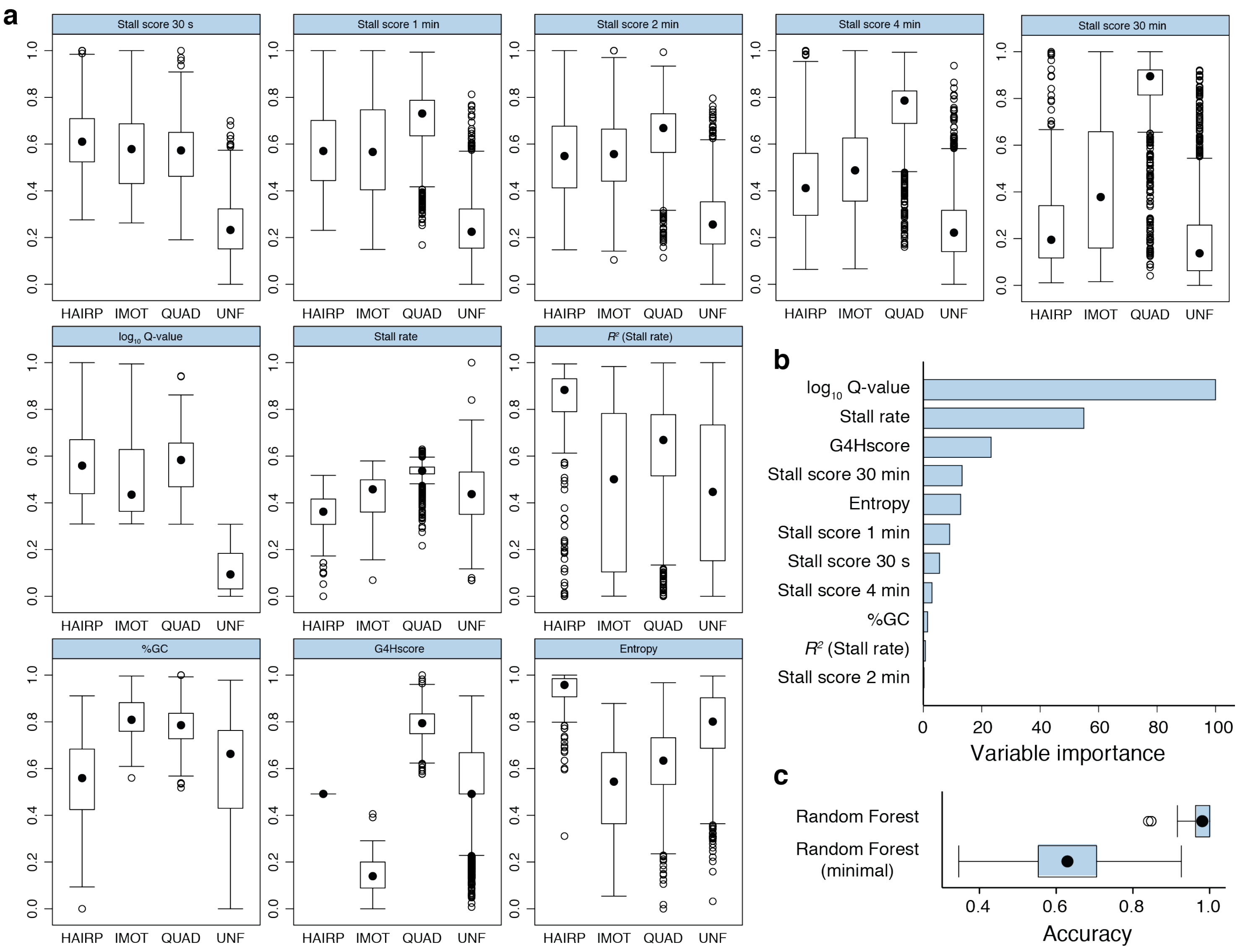
a
Stall score 30 s
Stall score 1 min
Stall score 2 min
Stall score 4 min
Stall score 30 min
log10 Q-value
Stall rate
R2 (Stall rate)
%GC
G4Hscore
Entropy
HAIRP IMOT QUAD UNF
b
log10 Q-value
Stall rate
G4Hscore
Stall score 30 min
Entropy
Stall score 1 min
Stall score 30 s
Stall score 4 min
%GC
R2 (Stall rate)
Stall score 2 min
Variable importance
c
Random Forest
Random Forest (minimal)
Accuracy

Figure S3. Supervised machine learning approach to structure prediction from DNA polymerase stalling events. (a) Distribution of values associated with the different features selected to train and select classifier algorithms to predict STR structures. The set of 2,932 control sequences was used to train, test and select the best performing algorithm. Each of the sequences was classified into one of the four structural classes HAIRP, IMOT, QUAD and UNF for hairpins, i-motifs, G4s and unfolded sequences respectively. UNF sequences comprised sequences whose stall scores are not statistically different from the scores of the negative control sequences, *i.e.* random sequences of varying GC content, at each time point. To identify those sequences, we assign to each stall scores a P value at each time point, using a Mann-Whitney U test challenging the replicate values against the distribution of values obtained for the negative control sequences, and combining these P values according to Fisher's method. Sequences with combined P values, referred to as Q values, higher than 0.1 were defined as UNF. The set of sequences used to train the classifiers then comprised 427 HAIRP, 105 IMOT, 983 QUAD and 1,417 UNF sequences. **(b)** Contribution of each feature to the selected random forest classifier indicating that the intensity of polymerase stalling and the kinetic of DNA synthesis are the most informative features in predicting the structure of the designed sequences. **(c)** We finally assessed whether features describing sequence composition, *i.e.* %GC, G4Hscore and sequence entropy, are sufficient for classifying structures. We then selected the best performing algorithm using these features only (Random Forest minimal) and found that while a random forest classifier using all features performed with an accuracy of 0.96 ± 0.03 over 100 resamplings, the minimal classifier performed with an accuracy of 0.64 ± 0.13 over 100 resamplings demonstrating that features describing sequence composition are not sufficient for structure prediction.

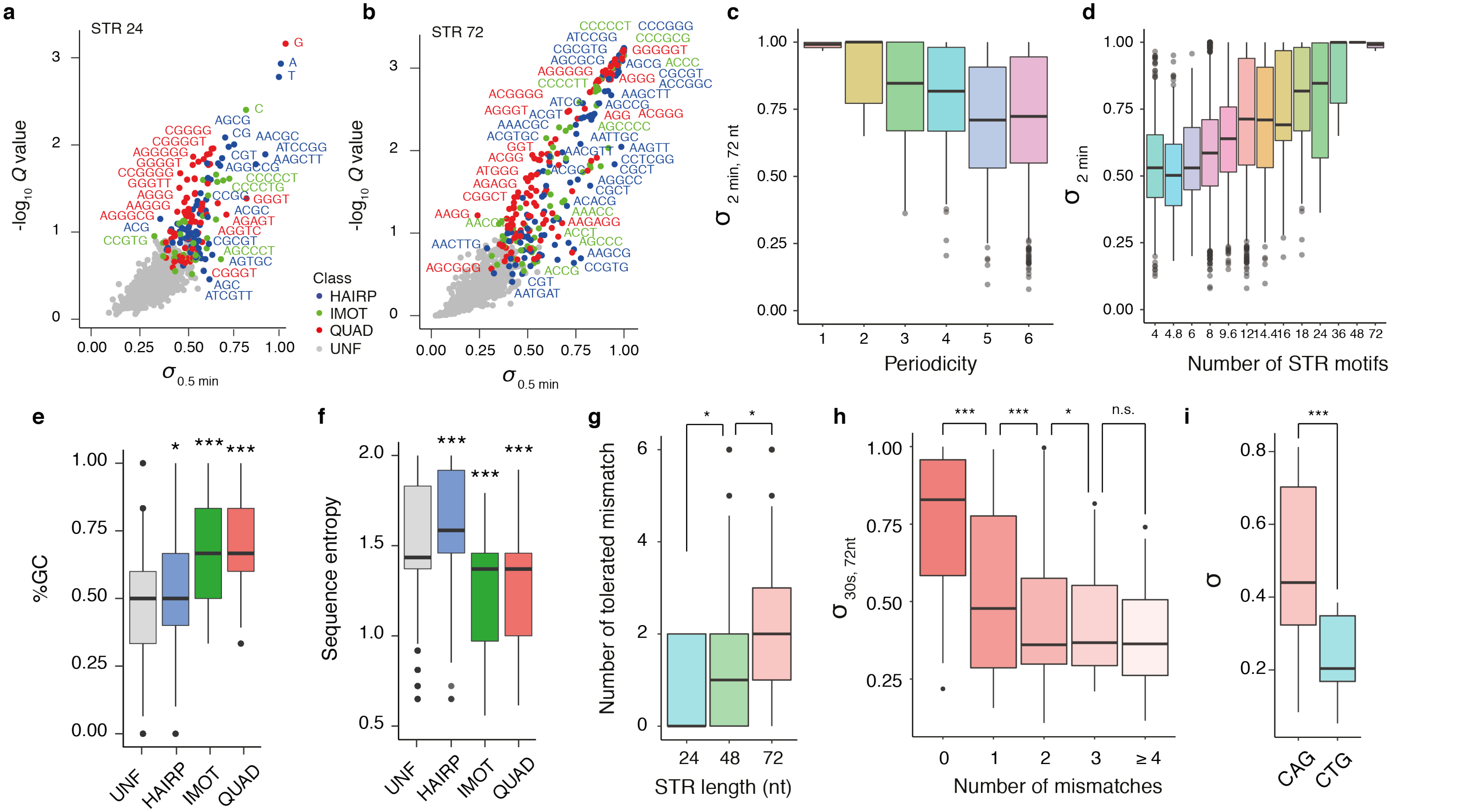

a
STR 24
-log10 Q value
σ 0.5 min
b
STR 72
-log10 Q value
σ 0.5 min
Class
HAIRP
IMOT
QUAD
UNF
c
σ 2 min, 72 nt
Periodicity
d
σ 2 min
Number of STR motifs
e
%GC
UNF
HAIRP
IMOT
QUAD
f
Sequence entropy
UNF
HAIRP
IMOT
QUAD
g
Number of tolerated mismatch
STR length (nt)
h
σ 30s, 72nt
Number of mismatches
n.s.
i
σ
CAG
CTG

Figure S4. Inferring STR structures from DNA polymerase stalling events. Stall scores of each 964 unique single-stranded STR motif together with their assigned structural classes when (**a**) 24 nt or (**b**) 72 nt long. Impact of STR periodicity (**c**) and number of STR motifs (**d**) on stall scores. (**e**) GC content and (**f**) sequence entropy of the different structural classes of 72 nt long STRs. Centre lines denote medians, boxes span the interquartile range, and whiskers extend beyond the box limits by 1.5 times the interquartile range. P values for the comparison of the distributions were calculated using Kolmogorov–Smirnov tests, $^{*}P \leq 0.05$, $^{***}P < 0.001$, and compare a given set of STR to the UNF STRs. (**g**) Number of tolerated mismatch in hairpin-like STRs. (**h**) Influence of the number of mismatches on the stall scores of hairpin-like STRs. (**i**) Stall scores associated with the CAG and CTG repeats. Centre lines denote medians, boxes span the interquartile range, and whiskers extend beyond the box limits by 1.5 times the interquartile range. P values for the comparison of the distributions were calculated using Kolmogorov–Smirnov tests, n.s. $P > 0.05$, $^{*}P \leq 0.05$, $^{***}P < 0.001$

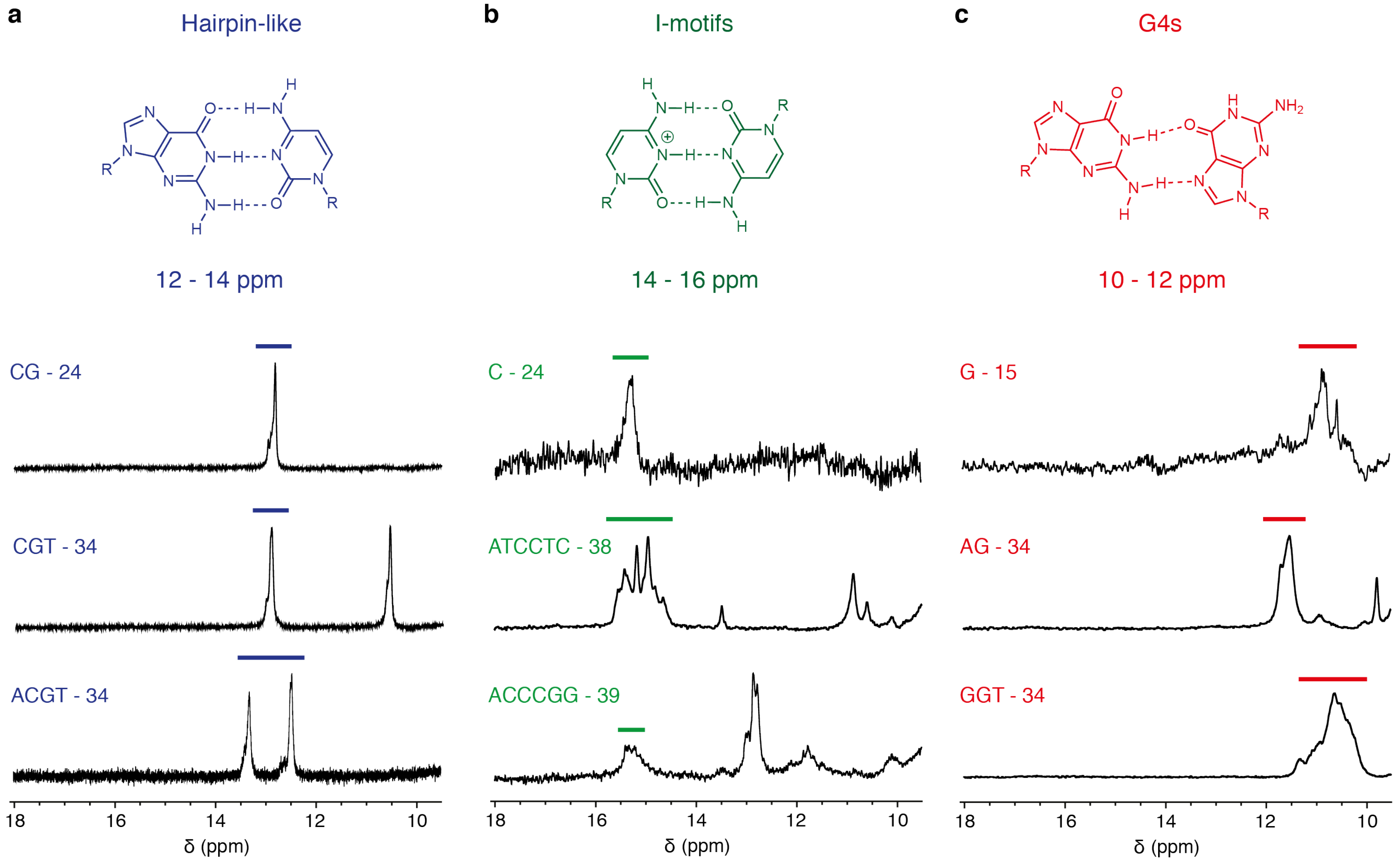

a
Hairpin-like
12 - 14 ppm
CG - 24
CGT - 34
ACGT - 34
b
I-motifs
14 - 16 ppm
C - 24
ATCCTC - 38
ACCCGG - 39
c
G4s
10 - 12 ppm
G - 15
AG - 34
GGT - 34
δ (ppm)

Figure S5. Validation of predicted STR structures. ^{1}H NMR spectroscopy was used to validate the structure of nine STRs. ^{1}H NMR was used to qualitatively probe the presence and the nature of imino protons involved in base pairing within three different classes of STRs: **(a)** hairpin-like, **(b)** I-motif and **(c)** G4 structures. Chemical shifts associated with imino protons can be used as a proxy for structure formation since hairpins, i-motifs and G4s are stabilised by Watson-Crick, hemi-protonated cytosine and Hoogsteen base pairing respectively. Such interactions are characterised by chemical shifts in the 12 to 14 ppm, 14 to 16 ppm and 10 to 12 ppm range respectively (Adrian et al., 2012; Esmaili and Leroy, 2005). Oligonucleotides were annealed at a final concentration of 0.2 mM in the same buffer used for the primer extension assay which is 40 mM Tris.HCl pH 7.5, 20 mM $MgCl_2$, 50 mM NaCl and 50 mM KCl. The exact sequences analysed are reported in Table S1. Coloured lines on top of NMR spectra highlights imino protons involved in Watson-Crick (blue), hemi-protonated cytosine (green) and Hoogsteen (red) base pairing respectively. Hence ^{1}H NMR allows confirming that the CG, CGT and ACGT repeats fold into hairpin-like structures, that the C, ATCCTC and ACCCGG repeats fold into i-motifs and that the G, AG and GGT repeats fold into G4s under the condition of our primer extension assay.

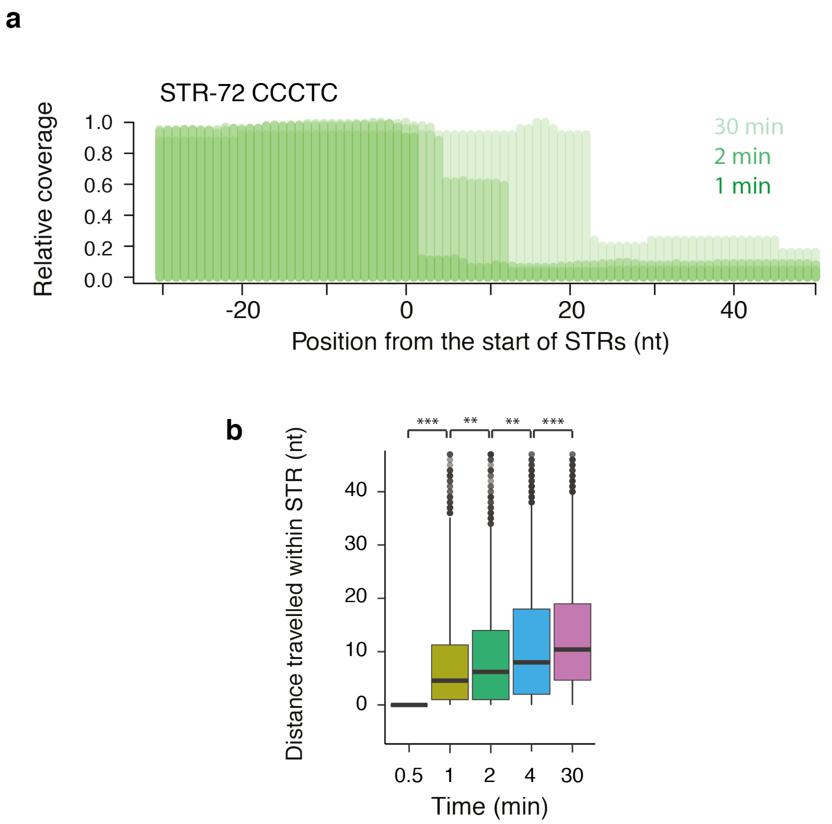

Figure S6. The DNA polymerase remodels STRs during DNA synthesis. (a) Relative read coverage across the 72-nt long CCCTC repeats, predicted to fold into an i-motif, highlighting time-dependent sharp transitions corresponding to the main positions where the DNA polymerase stalls and dissociates during DNA synthesis. **(b)** Distance travelled by the DNA polymerase within 72 nt long STRs over time. The positions of the stalled polymerase were defined as the position at which the relative coverage is equal at 0.5. In order to assess the distance travelled by the polymerase within the STRs, the difference between the positions of the stalled polymerase at a given time and the position at 0.5 min was considered. Centre lines denote medians, boxes span the interquartile range, and whiskers extend beyond the box limits by 1.5 times the interquartile range. *P* values for the comparison of the distributions were calculated using the Kolmogorov–Smirnov test, $^{**}P < 0.01$, $^{***}P < 0.001$.

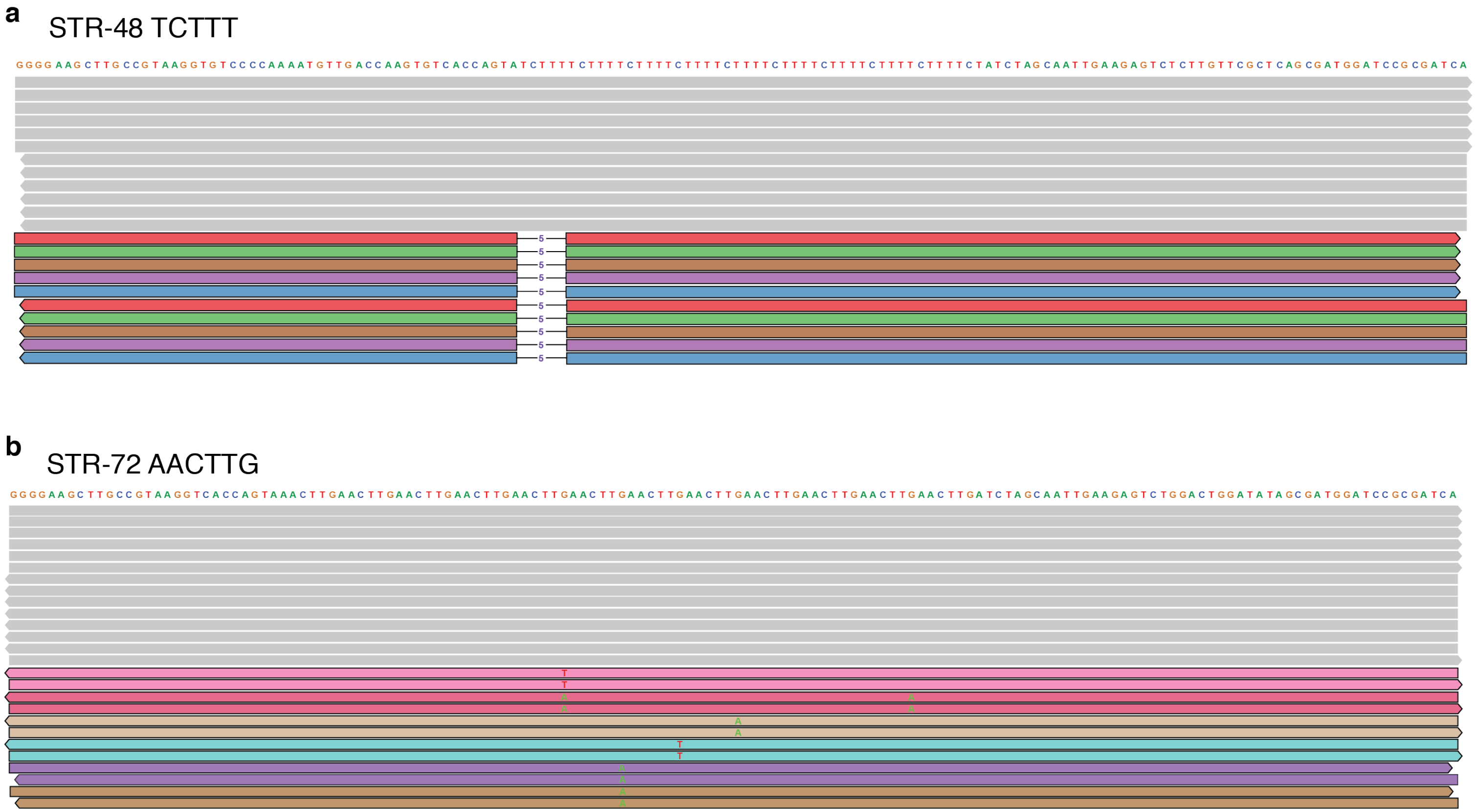

Figure S7. Identification of sequence variants within the pool of newly synthesised DNA molecules. Examples of STR alignment visualization in IGV. **(a)** Example of expansion events within the 48 nt long TCTTT repeat predicted to be unfolded under the condition of our primer extension assay. **(b)** Example of base substitutions called within the 72 nt long AACTGG repeat predicted to fold into a hairpin-like structure under the condition of our primer extension assay. While grey sequencing reads support the reference sequence, the coloured mate paired sequencing reads support the sequence variants. Reads were arbitrarily selected to represent sequence variant calling.

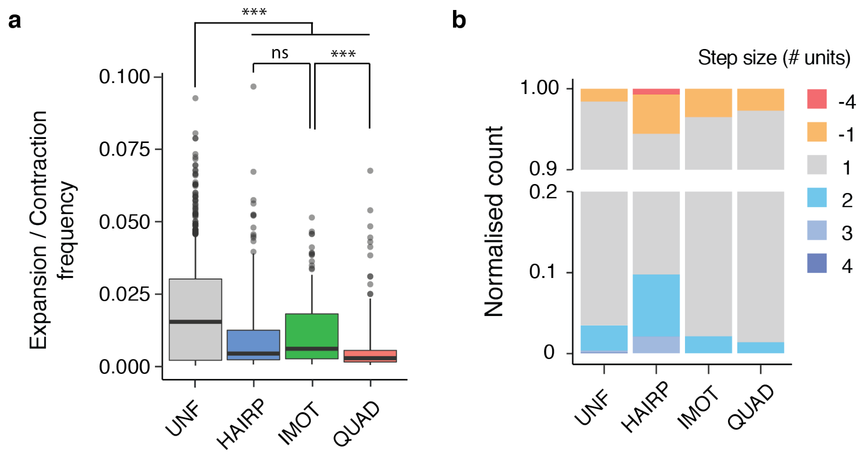

Figure S8. STR structures impact the frequency and nature of expansion/contraction events. (a) Frequency of expansion/contraction events at STRs when binned according to their structure. Frequencies are defined as the ratios of the number of reads supporting a mutation by the total number of reads covering this mutation. Centre lines denote medians, boxes span the interquartile range, and whiskers extend beyond the box limits by 1.5 times the interquartile range. P values for the comparison of the distributions were calculated using Kolmogorov–Smirnov tests, n.s $P > 0.05$, ***$P < 0.001$. **(b)** Step-size distribution of expansion/contraction events at STRs binned by structures. HAIRP STRs are more likely to mutate by multiple units at once and are more prone to contraction (odds ratio = 3.42; Fisher's two-sided $P = 1.39 \times 10^{-5}$).

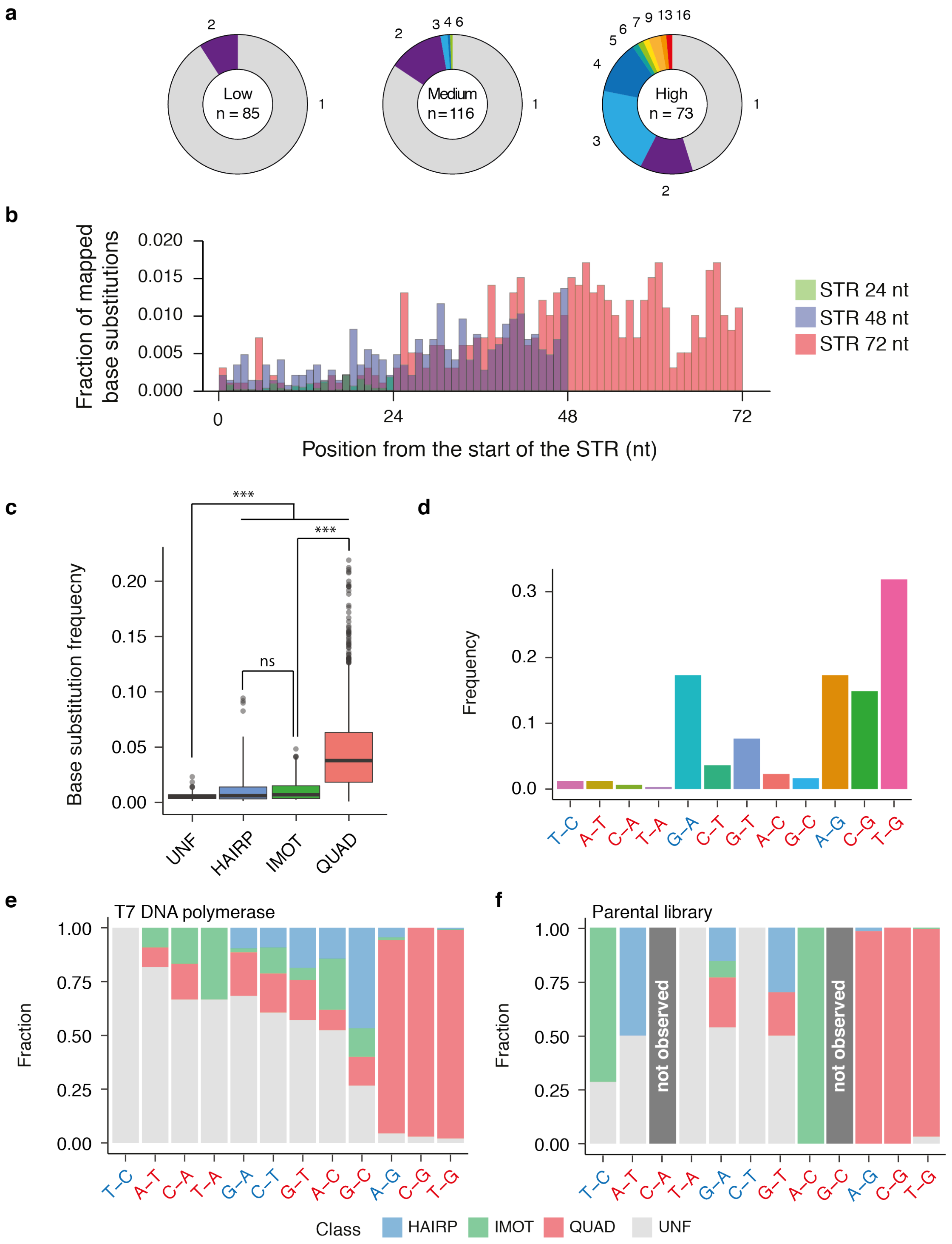

Figure S9. STR structures impact the frequency and nature of nucleotide substitution events. Nucleotide substitutions were called on the extended products at each time point and on the parental library, *i.e.* the library that has not been extended by the T7 DNA polymerase. To select *de novo* mutations, *i.e.* mutations arising from DNA synthesis by the T7 DNA polymerase rather than artefacts originating from the cloning, PCR and library preparation steps, any mutations called within the parental library were then excluded from the analysis. Analyses reported herein describe all the mutations called at any time points and within the repeats. **(a)** Proportion of

STR motifs carrying different numbers of point mutations when binned by their stall scores at 30 min (low ($\sigma < 0.33$), medium ($0.33 \leq \sigma < 0.66$) and high ($\sigma \geq 0.66$)). Segment sizes are proportional to the number of motifs carrying the number of mutations indicated around the periphery of the pie charts. The reported numbers of mutations are averages from individual sequences sharing the same motif. n is the total number of motifs considered for each analysis. **(b)** Fraction of base substitutions called within STRs of different length (24 nt, 48 nt and 72 nt long in green, blue and red respectively) highlighting an increased density of point mutations from the start to the end of the repeats. **(c)** Frequency of base substitution events at STRs when binned according to their structure. Frequencies are defined as the ratios of the number of reads supporting a mutation by the total number of reads covering this mutation. Centre lines denote medians, boxes span the interquartile range, and whiskers extend beyond the box limits by 1.5 times the interquartile range. p-values for the comparison of the distributions were calculated using Kolmogorov–Smirnov tests, n.s $P > 0.05$, $^{***}P < 0.001$. Frequency **(d)** and representation **(e)** of each possible nucleotide substitution events observed within STRs of different structural classes. The representation of each possible nucleotide substitution events called within the parental library is shown in **(f)** for comparison. These observations show that specific mutation patterns are associated with each structural class of STRs and are mainly due to nucleotide misincorporation by the T7 DNA polymerase. Nucleotide substitution preferences, *i.e.* substitution matrixes, relative to each structural class are reported in Figure S10.

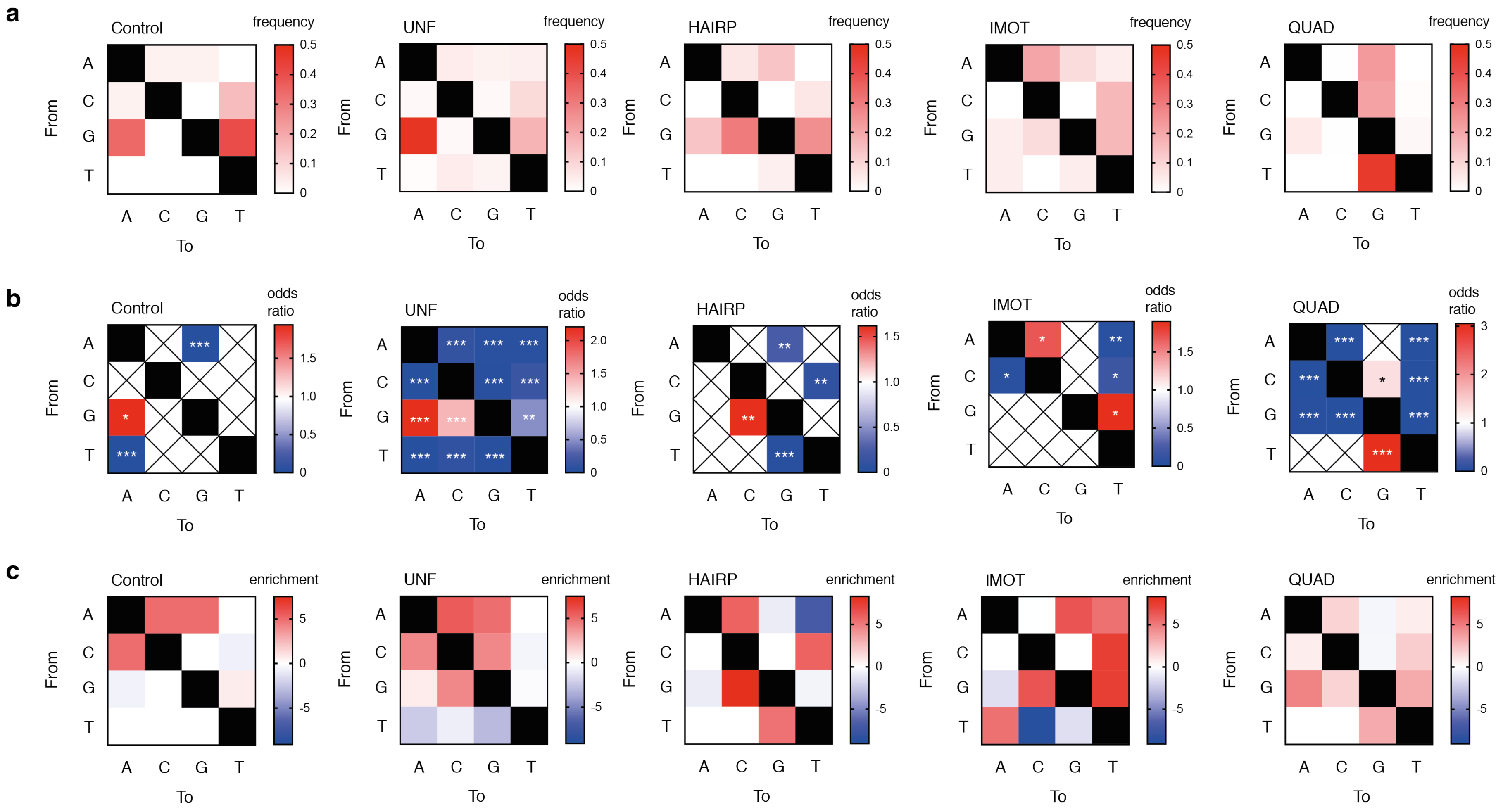

a
Control
UNF
HAIRP
IMOT
QUAD
frequency
From
To
A
C
G
T
b
odds ratio
***
**
*
c
enrichment

Figure S10. Structure-dependent nucleotide substitution preferences. (a) Substitution matrixes associated with each structural class of STR describing preferences in nucleotide misincorporation by the T7 DNA polymerase. To select mutations arising from DNA synthesis by the T7 DNA polymerase rather than artefacts originating from the cloning, PCR and library preparation steps, any mutations called within the parental library, *i.e.* the library that hasn't been extended by the T7 DNA polymerase, were excluded from the analysis. Control sequences refer to the set of 1,000 random sequences of varying GC content and their associated matrix describe the basal bias in nucleotide substitution. The substitution matrix associated with the unfolded (UNF) STR motifs is very similar showing that the T7 DNA polymerase nucleotide misincorporation preference is not affected by repeated sequences. On the other hand, substitution matrixes associated with STR motifs prone to hairpin (HAIRP), i-motif (IMOT) and G4 (QUAD) formation are distinct from those associated with the control sequences and UNF motifs. Moreover, each structural class displays distinct substitution matrixes suggesting that they induce unique mutational signatures. For example, N to G substitutions, *i.e.* misincorporation of C by the T7 polymerase, is found almost exclusively within STRs folding into G4s (odds ratio = 12.04; Fisher's two-sided $P < 2.2 \times 10^{-16}$). Similarly, G to C (odds ratio = 25.43; Fisher's two-sided $P = 1.89 \times 10^{-6}$) and G to T (odds ratio = 14.35; Fisher's two-sided $P = 6.67\times 10^{-4}$) substitutions are enriched within the HAIRP and IMOT STRs respectively. We then assessed whether these unique mutational signatures could be explained by biases in base composition. To do this, we tested the differences between the observed frequencies and the expected frequencies from the underlying base composition of the STR motifs using Fisher's two-sided tests. Panel **(b)** reports the odds ratios associated with the frequencies of each nucleotide substitution events together with their associated *P* values. Hence, events in red and blue are events that are observed more or less frequently than expected by chance considering only the base composition of the STR sequences respectively. *$P \leq 0.05$, **$P < 0.01$, ***$P < 0.001$, non-significant values have been omitted for clarity. In order to assess whether these unique mutational signatures are exclusive to the T7 DNA polymerase, we computed the enrichment of the frequency of each mutation events in the extended products from their frequency in the parental library. Panel **(c)** reports substitution matrices in which the reported values are the $\log_2$ fold enrichment of frequencies. Such representation allows highlighting T7 DNA polymerase associated nucleotide substitution preferences.

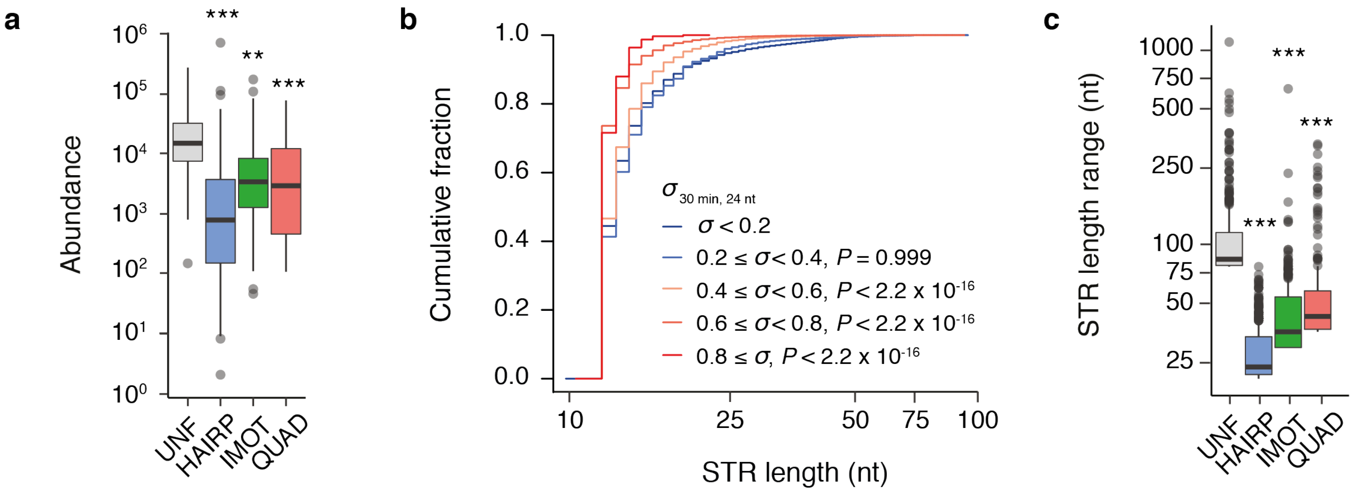

Figure S11. DNA polymerase stalling at DNA structures predicts abundance and length of STRs in the human genome. (a) Abundance, *i.e.* the number of occurrences in the human genome of the 501 unique double-stranded STR motifs in the human genome when binned by their structural class. **(b)** Cumulative distribution of STR length in the human genomes binned by their stall scores at 30 min when 24 nt long. **(c)** Range of double-stranded STR motifs length when binned by their structural class. The plot reports the top 1,000 longest repeat instances from each bin. In box plots, centre lines denote medians, boxes span the interquartile range, and whiskers extend beyond the box limits by 1.5 times the interquartile range. P values for the comparison of the distributions were calculated using Kolmogorov–Smirnov tests, $^{**}P < 0.01$, $^{***}P < 0.001$. For panels **(a)** and **(c)**, p-values compare each structural class to the distribution observed for unfolded (UNF) STRs. For panel **(b)**, P values compare each distribution to the bin of immediate lower stall score value.

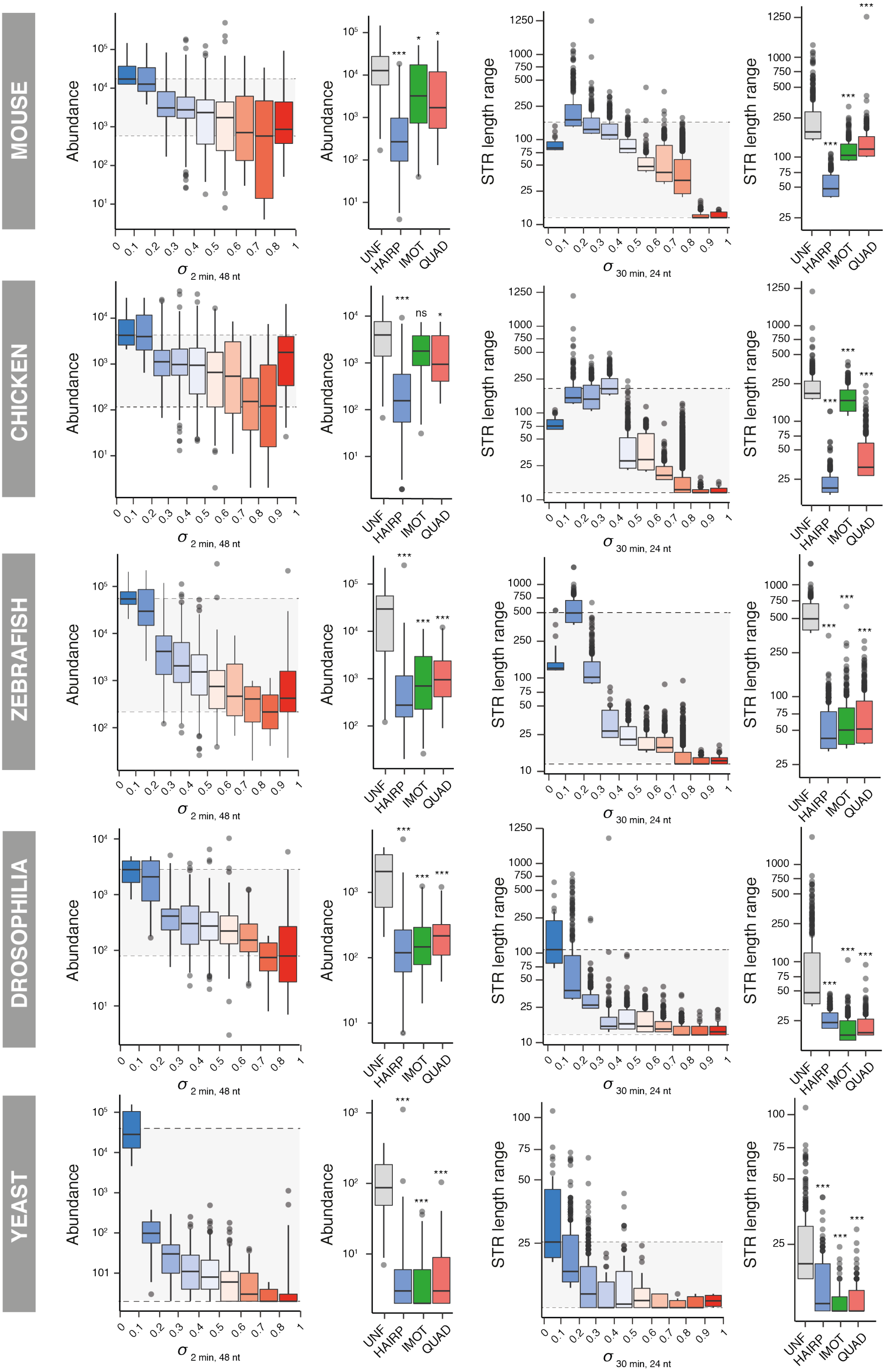
MOUSE
CHICKEN
ZEBRAFISH
DROSOPHILA
YEAST
Abundance
STR length range
σ 2 min, 48 nt
σ 30 min, 24 nt
UNF
HAIRP
IMOT
QUAD
***
*
ns

Figure S12. DNA polymerase stalling at DNA structures predicts abundance and length of STRs in eukaryotic genomes. Abundance, *i. e.* number of occurrences, of the 501 unique double-stranded STR motifs in five eukaryotic genomes when binned by their average stall scores at 2 min when 48 nt long or their structural class. Range of double-stranded STR motifs length when binned by their average stall scores at 30 min when 24 nt long or their structural class. The plots report the top 1,000 longest repeat instances from each bin. The analysed genomes were *Mus musculus* (mm10), *Gallus gallus* (galGal6), *Danio rerio* (dm6), *Drosophila melanogaster* (dm6) and *Saccharomyces cerevisiae* (sacCer3). *P* values for the comparison of the distributions were calculated using Kolmogorov–Smirnov tests, n.s $P > 0.05$, *$P < 0.05$, ***$P < 0.001$.

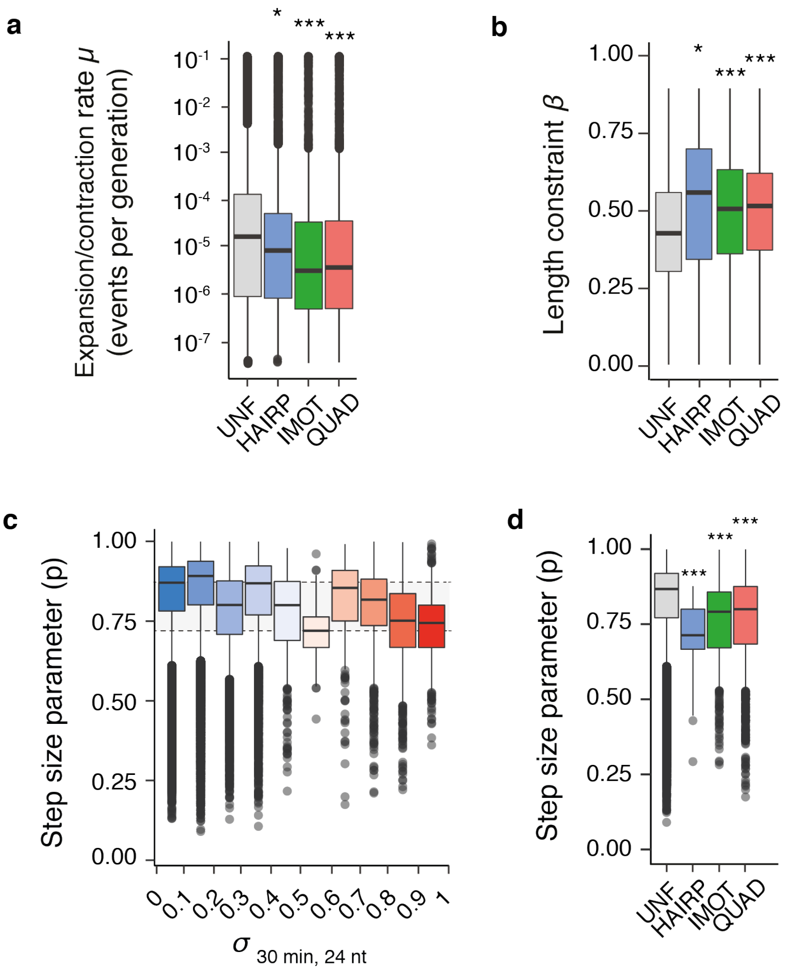

Figure S13. DNA polymerase stalling at DNA structures predicts STR stability in the human genome. (a) Expansion/contraction rates (μ) and **(b)** strength of the directional bias of mutation (β) of the double-stranded STR motifs when binned by their structural class. Step size parameter (p), *i.e.* the probability that a mutation occurs at a single STR unit at a time, associated with each double-stranded STR motifs when binned by **(c)** their average stall scores at 30 min when 24 nt long or **(d)** their structural class. P values for the comparison of the distributions were calculated using Kolmogorov–Smirnov tests, $^*P \leq 0.05$, $^{***}P < 0.001$. P values compare each structural class to the distribution observed for the unfolded (UNF) STRs.

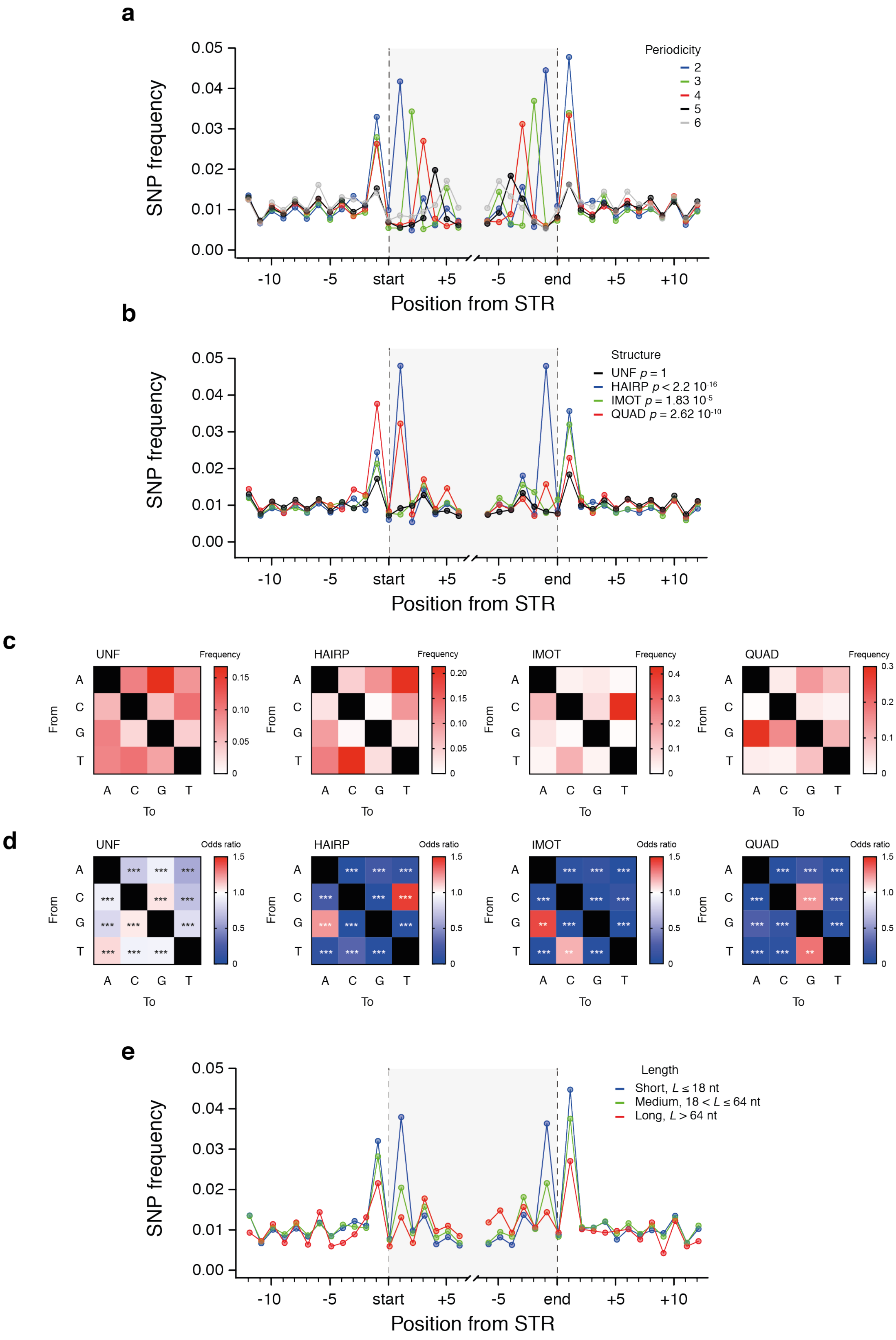
a
SNP frequency
0.05
0.04
0.03
0.02
0.01
0.00
-10
-5
start
+5
-5
end
+5
+10
Position from STR
Periodicity
2
3
4
5
6
b
Structure
UNF $p = 1$
HAIRP $p < 2.2\ 10^{-16}$
IMOT $p = 1.83\ 10^{-5}$
QUAD $p = 2.62\ 10^{-10}$
c
UNF
HAIRP
IMOT
QUAD
Frequency
From
To
A
C
G
T
d
Odds ratio
e
Length
Short, $L \leq 18$ nt
Medium, $18 < L \leq 64$ nt
Long, $L > 64$ nt

Figure S14. Structured STRs are prone to sequence variation in the human genome. SNP density at positions surrounding STRs when binned according to their **(a)** periodicity, **(b)** structure or **(e)** length. SNP density at individual positions was computed by dividing the number of STR marked by a SNP at a given position by the total number of considered STRs. Panel **(a)** shows a periodic pattern in SNP enrichment in the vicinity of STRs that reflects the repetitive nature of STRs. Panel **(b)** highlights structure-dependent sequence variation and suggest error-prone DNA synthesis at DNA structures. Reported *P* values assess SNP enrichment in the vicinity of STRs and were computed using two sided Fisher tests considering the average frequency of SNPs at position -1 and +1 from the start and the end of the repeats. **(c)** Structure-specific substitution matrices were built by considering nucleotide substitution preferences at internal positions of the repeats (from the start to start + 6 nt and from the end - 6 nt to the end of the repeats). These matrices suggest that each structural class of STRs is associated with a specific mutation signature within the human genome as observed *in vitro* with our primer extension assay (see Figure S10). While the matrices obtained from SNP distributions in the human genome and from our primer extension assay differ, they are unique for each structural class. Panel **(d)** reports the odds ratios and associated *P* values obtained by testing the differences between the observed frequencies and the expected frequencies from the underlying base composition of the STR motifs using Fisher's two-sided tests (as in Figure S10B). $^{**}P < 0.01$, $^{***}P < 0.001$.

Table S1. Oligonucleotides used for the validation of STR structures by ^{1}H NMR spectroscopy.

Sequence name	Sequence
CG-24	TCGCGCGCGCGCGCGCGCGCGCGT
CGT-34	TCGTCGTCGTCGTCGTCGTCGTCGTCGTCGTCGT
ACGT-34	CGTACGTACGTACGTACGTACGTACGTACGTACG
C-24	TCCCCCCCCCCCCCCCCCCCCCCT
ATCCTC-38	CCTCATCCTCATCCTCATCCTCATCCTCATCCTCATCC
ACCCGG-39	CCCGGACCCGGACCCGGACCCGGACCCGGACCCGGACCC
G-15	TTGGGGGGGGGGGGGGGT
AG-34	TGAGAGAGAGAGAGAGAGAGAGAGAGAGAGAGAT
GGT-34	TGGTGGTGGTGGTGGTGGTGGTGGTGGTGGTGGT

Supplementary references

Adrian, M., Heddi, B., and Phan, A. T. (2012). NMR spectroscopy of G-quadruplexes. Methods *57*, 11-24.

Bedrat, A., Lacroix, L., and Mergny, J. L. (2016). Re-evaluation of G-quadruplex propensity with G4Hunter. Nucleic Acids Res *44*, 1746-1759.

Esmaili, N., and Leroy, J. L. (2005). i-motif solution structure and dynamics of the d(AACCCC) and d(CCCCAA) tetrahymena telomeric repeats. Nucleic Acids Res *33*, 213-224.

Marmur, J., and Doty, P. (1962). Determination of the base composition of deoxyribonucleic acid from its thermal denaturation temperature. J Mol Biol *5*, 109-118.
