# Supplementary material for: DNA polymerase stalling at structured DNA constrains the expansion of short tandem repeats
Source: Genome Biol. 2020 Aug 21;21:209. doi: 10.1186/s13059-020-02124-x (PMC7441554; doi:10.1186/s13059-020-02124-x)
Supplement: Supplementary file 3 — Additional file 3. Review history. [file 13059_2020_2124_MOESM3_ESM.docx]

Review History

**First round of review**

**Reviewer 1**

**Are you able to assess all statistics in the manuscript, including the appropriateness of statistical tests used?**

No, I do not feel adequately qualified to assess the statistics.

**Comments to author:**

In the work "DNA polymerase stalling at structured DNA constrains the expansion of Short Tandem Repeats" the authors present a comprehensive study looking at whether T7 polymerase will stall at a variety of short tandem repeat sequences in order to understand the mechanisms of repeat variation and stability in genomes. The authors utilize a library approach where they design oligos that contain over ~5000 permutations of short tandem repeats that range from 1-6 nucleotides in length and then utilized these sequences in their novel high-throughput in vitro polymerase extension assay. Utilizing this assay they unveil several interesting points on the nature of structure forming repeats. First, they conclude that most STRs in their library are able to eventually be extended by T7 polymerase, suggesting that DNA polymerase stalls at STRs are generally able to be overcome. Next, mostly turning their focus on DNA hairpins and G-quadruplex structures, they show that DNA hairpins and G4 structures have distinct properties. They show that G4 structures are most likely to result in persistent polymerase stalling and have a high percentage of base substitutions while hairpin forming sequences are likely to be fully replicated by the polymerase but experience contraction events and to mutate by multiple units at once. The authors do additional comparative analysis where they compare findings to the human genome and postulate how the nature of the structure can contribute to genome instability. Altogether, this study adds significant new understanding of how polymerase stalling at DNA structures is modulated by the length and type of DNA structure and how this influences mutations of microsatellites in genomes. It is generally a well-written and presented study, with appropriate controls and statistical analysis.

Major comments

1. What about triplex structures? These are not addressed (not mentioned and no controls designed).

2. Are the high-scoring hairpins basically all the palindromic sequences (that can form a hairpin without mismatches)? Were there any other characteristics that came out of the analysis - what was learned about this class?

3. How did you determine the ideal number of nucleotide lengths to test? Along that line, one part of this study that is largely ignored is how many bases participate in the repeat which may impact the size of the overall structure/barrier (for example, for the 72nt example, 1 nt repeat would equal 72 repeat units, 2nt=36 repeats, etc). If anything, if your longer STRs have produce a stronger stall it's more impressive because the overall number of structures that can form are fewer. Similarly, shorter STRs have more base pairing opportunities/ perhaps not all repeats are part of the structure so perhaps it's not so surprising that your polymerase travels more internal initially before stalling. In your analysis, did you uncover any relationship between STR unit length and stalling?

4. Figure 2F. Your statistics are all relative to the repeat sequences and not the control. For example, are i-motif's different from the control? In p8 ln7-11 you infer that this mathematical calculation will allow you to separate out different sequences but if one of them isn't statistically different from the control, is this really true? Is the library different from the control? It looks like the median is like the control and the outlying hairpin and G4 sequences are what drive differences.

5. P8 ln38-42. You do not show the control data or statistics to illustrate that UNF sequences are not different from control sequences. From what I can determine, you are calling these unfolded sequences because they aren't different from the control in your assay. Is this correct? These are still STR sequences, so is it possible that they are structured but missed by the constraints of your assay? I presume your control sequences aren't predicted to form secondary structures (are truly random). Are the UNF sequences predicted to form structures? It doesn't seem appropriate to utilize the UNF sequences for comparison statistics when comparing UNF, HAIRP, IMOT, and QUAD like in figure 3b, 3c, 3d, and 3e.

6. It is not clear what the sequences of your G4 and hairpin motif's are in all of your experiments. Are all your control sequences what are listed in figure legend 2A and 2B? They aren't tandem repeats—the G4 sequence is 32 nucleotides, which isn't a length you test in your library. Additionally, it seems to have only (4) G4 sequences - a tandem repeat would have 8. The hairpin sequence would also form only 1 large hairpin - an STR could form many smaller hairpins which may present differently in your assay. Using these as a control which you compare things to may skew your results. Please clarify in text what the control sequences are, why they are ideal controls for this study and what, if any, are the limitations of these controls.

7. Need to qualify and discuss some conclusions as you have only measured one aspect of STR replication, namely how structures in the template strand affect polymerase progression. There are many others not measured here. For example, the bias to contractions when replicating hairpins is not surprising, as it has been long known that they occur by structure formation on the template strand. In contrast, expansions usually occur by structure formation on a displaced non-template strand, something not present in this system.

Minor comments (items to address):

1. P4, Ln 38-42. Some evolutionary context may be helpful here. You're using a library approach initially that then ties into human genomes more generally. It would be helpful to understand how T7 DNA polymerase models Eukaryotic/mammalian replication.

2. Figure 1b and pg 6 ln15-20. What are these transient replication stalls? Could you label them in your figure? To show these are replication stalls and not just non-specific bands could you show a t=0/pre-incubation with T7 lane?

3. Figure 2a. What is represented in each bar? What is the Y-axis?

4. Figure 2c. You don't really discuss figure 2c in the text other than you ran the library against G4 and hairpin controls—What does the data mean? The median stall score? Does it change? What do you make of the "significant?" population that has a 100% stall rate for all timepoints?

5. Pg 7, Fig 2. Lambda () is not well defined. Please add a more complete description of what this parameter represents and how it was calculated.

6. I suggest you remove HAIRP, QUAD, IMOT (and UNF) abbreviations in the text and just call them hairpins, quadruplexes and i-motifs for better readability.

7. Fig. 3a. Why was sigma at 0.5 min chosen (x axis, explain logic)? What is the Q value on the Y axis and how is that related to the stall score? This graph and how to read it should be explained better to the reader.

8. Pg 12 lines 52-55 should read "Taken together these observations suggest that STR mutational profile is intimately linked to polymerase stalling and to STR length and sequence stability."

**Reviewer 2**

**Are you able to assess all statistics in the manuscript, including the appropriateness of statistical tests used?**

No, I do not feel adequately qualified to assess the statistics.

**Comments to author:**

The human genome contains over 4 million Short Tandem Repeats (STRs). While expansion of several STRs have been linked to pathological consequences, how STRs are constrained is poorly understood. In this manuscript by Murat et al., the authors investigate how STRs affect DNA synthesis to determine if this can explain their instability. For this, they generated a ssDNA plasmid library that contains about 20 thousand STRs covering all permutations of 1-6 nt repeats with three defined lengths. By replicating the STRs with T7 DNA polymerase, they assigned each STR into four classes (unstructured, hairpin, i-motif, and G4) based on how their replication kinetics compares to STRs known to fold into these four classes. They then sequenced the replication products and find that structured STRs are more prone to point mutations, while unstructured STRs tend to induce polymerase slippage leading to expansions/contractions. Interestingly, they also find that structured STRs are significantly shorter and less abundant compared to unstructured STRs in eukaryotic genomes including the human genome. This is likely driven by the higher degree of point mutations in structured STRs.

This is a well-executed study that provides important information on the evolution and maintenance of repeats in our genome. It is somewhat surprising that the simplified system used in this study (primer extension on ssDNA templates by a T7 polymerase) can generate information relevant to the in vivo situation. However, the data is consistent with several aspects of repetitive sequences in our genome and this system can therefore have predictive value. Although this is a new and powerful approach and fits well with this journal, there are some issues that need to be addressed before I would recommend final acceptance.

Major points:

1. How can the authors exclude that the PCR amplification of the stalled and extended products introduces PCR bias. The stalled products can be a lot smaller compared to the extended products and 35 cycles of PCR could favor amplification of these smaller products which could have a big effect on the stalling factor. Is there any evidence that this does not occur? this should at least be addressed.

2. While the data generated by primer extension with the engineered T7 DNA polymerase (Sequenase) correlates with several aspects of STRs in our genome, this polymerase differs in important aspects from the mammalian replicative polymerases: it lacks exonuclease activity and it is an A-family polymerase and not a B-family polymerase. The results would gain a lot of strength if a similar assay would be performed with a B-family polymerase, at least on a selection of STRs, and similar data would be obtained.

3. In the STR library, inter-plasmid (STR) annealing might occur during heating and cooling down of the plasmids, raising the possibility of incorrect stalling scores. Is it possible to take into account the inter-STR annealing for STR classification, ie by eliminating complimentary sequences from structured STRs etc?? This possibility needs to be discussed at least.

4. Both the positive control STRs that are known to fall into one of the four structural classes, and the STRs predicted to fold into these structures are referred to as ' unstructured, hairpin, i-motif, and G4'. As for most of the 'random' STRs, as it has not been experimentally shown that they fold into these structures (this is only based on the parameters determined by the data), it would be more correct to refer to these as I-motifpred, G4pred etc. (or something along these lines). This would also make this manuscript easier to follow (e.g. it would show that the data used for Fig 2a/6 and 3 is different).

Minor points:

- In two places (p9 and p16), the condition of the assay is referred to as 'physiological condition'. However, the condition greatly from a cellular context, this should be rephrased.

- Figure 1b: What is the fully extended product? It is not clear which band the arrow points at. If the G4 fully stalls replication, why is there a band at the height of the extended band? Or is this not the fully extended product? In addition, how are the stalled bands isolated. For the G4, this is a single band but this experiment is performed in pools to the stalled band is likely a smear. This should be explained.

- Although the stall score seems to be a powerful parameter, it is very difficult to understand exactly how it is calculated. In the method section the following is writing: 'Stall scores were then computed as the ratio of the number of reads associated with a sequence in the stall fraction over the total number of reads associated to the same sequence in both the stalled and extended fractions'. How is this made relative to all other sequences in the library (mentioned on page 7)? How can this score go up for the G4 structures. This needs to be explained better.

- Figure 2d: The example for the G4 structure is very different from the average based on 2a, this makes 2d difficult to understand. Could a more representative example be taken?

-Figure 2f: Why does the i-motif structures display positive lambda values, while they are likely to be resolved like hairpin (Figure S2a and b). This should be explained in the text. Related to this, figure S2g-I. If I understand correctly, lower lambda value means quicker synthesis after stalling. Why do hairpins with higher Tm show lower lambda values in panel g? Moreover, why do G4 and imotif structures show predominantly negative lambda values but it is concluded that these structures have positive lambda?

- Figure 3a: Is there any correlation with known pathological STRs and the authors' structure assignment or stalling scores. This would be interesting to mention.

- Figure 4d: The color scheme is confusing, blue/pink is also used for hairpin/G4 structure in the rest of the figure.

- p13: 'suggesting that STRs capable of significant secondary structure are deleterious. Indeed, structured STRs are less abundant that their unstructured counterparts'. Yes, but G4s are more abundant than hairpins even though they are more structure. Therefore, this correlation does not fit exactly. How is this explained? See also in figure S13.

- Related to this, figure 4e: The authors argue that structured motifs are enriched in the pool of STRs exhibiting point mutation, but actually only G4 structures appear to be enriched as shown in Figure S9c. To make this point clear, it is better to compare the point mutation frequency among UNF and other respective structures like Figure S8a. Also Figure S8a should have UNF to directly compare slippage frequency between unstructured STR and structured species.

- Figure 5f: The authors state that "the higher the stall score of a STR, the higher the density of SNPs at the boundary of the locus", but actually is there significant difference between medium and high sigma values? This point should be clarified.

- Several supplementary Figures are not referred to in the main text.

**Reviewer 3**

**Are you able to assess all statistics in the manuscript, including the appropriateness of statistical tests used?**

Yes, and I have assessed the statistics in my report.

**Comments to author:**

This is an elegantly simple and powerful study of a DNA polymerase stalling behavior of all possible STR with very clear results and conclusions supported by adequate statistical analysis. The limits of the study are nevertheless clearly stated and discussed, mostly in the discussion. This is one of the rare times where I have no serious criticism or pressing question about a manuscript.

Still, it is not clear to me to which point the behavior of T7 DNA polymerase can be taken to reflect the behavior of the replicative DNA polymerases in the organisms studied, whether in terms of stalling, slippage or error-prone DNA synthesis. The implicit reasoning of the authors is that such events in their deliberately simple in vitro system are sufficiently similar to the in vivo DNA replication process to explain the observed size and abundance distributions and SNP densities around simple repeats. This is probably true, given the striking correlations observed, but some mechanistical justification based on existing literature would be welcome if there is any beyond the authors' observations.

This work is rather unique and potentially very useful to researchers in the field. I would have no objection to accept this manuscript especially if they can answer my single concern above.

**Authors Response**

**Point-by-point responses to the reviewers’ comments:**

*We thank the reviewers for their helpful comments and suggestions.*

*The three reviewers commented on the use of the T7 DNA polymerase (Sequenase) in our primer extension assay and reviewer 2 suggested that the study of a B family polymerase would strengthen our manuscript. We offer here a thorough explanation for why we selected the T7 DNA polymerase for our assay:*

*(i) The main objective of our work was to develop a high throughput assay that allows monitoring of the behaviour of a model polymerase at each STR permutation and use this in vitro data for explaining the evolution of STR in eukaryotic genomes. Because VNTRs and SNPs at STR loci within eukaryotic genomes are the consequences of the accumulation and fixation of rare events, we selected an exonuclease deficient polymerase in order to detect within the course of a single round of DNA synthesis all possible mutational events. The Sequenase polymerase is a genetically engineered form of T7 DNA polymerase that has virtually no proofreading/exonuclease activity while maintaining the high processivity of the parental polymerase (S Taboret al. J. Biol. Chem. 1989, 264, 6447-6458).*

*(ii) STRs within eukaryotic genomes are replicated according to either leading- or lagging-strand DNA synthesis mechanisms. Both mechanisms greatly differ as they involved different polymerases, i.e. Pol α, Pol δ and Pol ε, with different features such as ribonucleotide usage, processivity, fidelity and proofreading activity. Each polymerase is associated with different mutational spectrums and rates. Because, we can’t know the replication polarity of each STR within each eukaryotic genome analysed, we needed a polymerase known to perform both leading and lagging strand synthesis in order to analyse global replication biases rather than strand specific effects. In this context we selected the T7 DNA polymerase as it carries out both leading- and lagging-strand DNA synthesis (Gao et al. Science 2019, 363, eaav7003).*

*(iii) In order to assess the impact of DNA structures to DNA synthesis at STRs, it is crucial to use a polymerase that will stall due to structure formation rather than the repeated nature of the DNA template. Previous work has demonstrated that, due to its highly processive nature, the T7 DNA polymerase (Sequenase) stalls preferentially at STR structures (Kang et al. J. Biol. Chem. 1995, 270, 27014–27021; Ohshima et al. J. Biol. Chem. 1997, 272, 16798–16806). The polymerase catalytic cores of A and B family polymerases are broadly similar in their architecture (Johansson et al. Cold Spring Harb. Perspect. Biol. 2013, 5, a012799) but multisubunit mammalian B family replicative polymerases, will require additional co-factors, such as PCNA, to perform with similar processivity, significantly complicating the conditions of the assay.*

*For all these reasons, we feel that the T7 DNA polymerase (Sequenase) represented the best compromise for use in our high throughput primer extension assay in order to understand the evolution of structured STRs. We have clarified this point in the manuscript (P6 L6-12, P19 L15-23). We appreciate that studying how B family polymerases behaves at STRs is of great interest, but we anticipate that the main conclusion of the paper won’t be either affected nor strengthen as the catalytic core and the DNA polymerase channel of the A and B family of polymerases are well conserved (Wu et al. Chem. Rev. 2014, 114, 2759−2774). We are currently optimizing our assay for studying the role of replicative accessory factors in replicating STRs using reconstituted replication forks from purified proteins. The results from this work will be reported in due course.*

Reviewer #1:

In the work "DNA polymerase stalling at structured DNA constrains the expansion of Short Tandem Repeats" the authors present a comprehensive study looking at whether T7 polymerase will stall at a variety of short tandem repeat sequences in order to understand the mechanisms of repeat variation and stability in genomes. The authors utilize a library approach where they design oligos that contain over ~5000 permutations of short tandem repeats that range from 1-6 nucleotides in length and then utilized these sequences in their novel high-throughput in vitro polymerase extension assay. Utilizing this assay they unveil several interesting points on the nature of structure forming repeats. First, they conclude that most STRs in their library are able to eventually be extended by T7 polymerase, suggesting that DNA polymerase stalls at STRs are generally able to be overcome. Next, mostly turning their focus on DNA hairpins and G-quadruplex structures, they show that

DNA hairpins and G4 structures have distinct properties. They show that G4 structures are most likely to result in persistent polymerase stalling and have a high percentage of base substitutions while hairpin forming sequences are likely to be fully replicated by the polymerase but experience contraction events and to mutate by multiple units at once. The authors do additional comparative analysis where they compare findings to the human genome and postulate how the nature of the structure can contribute to genome instability. Altogether, this study adds significant new understanding of how polymerase stalling at DNA structures is modulated by the length and type of DNA structure and how this influences mutations of microsatellites in genomes. It is generally a well-written and presented study, with appropriate controls and statistical analysis.

Major comments

1. What about triplex structures? These are not addressed (not mentioned and no controls designed).

*Our assay uses primed single-stranded DNA templates as substrates for DNA synthesis and we thus focussed on DNA structures arising within single-stranded DNA. Single-stranded repeats can only fold into hairpin-like and tetrahelical structures (G-quadruplexes and I-motifs). While triplexes, or H-DNA, are known to impede replication (reviewed in Mirkin, Nature, 2007, 447, 932-940), they form in repeats within double-stranded DNA, or require RNA. Hence, the effect of triplexes on DNA replication cannot be monitored using our assay. In fact, a well characterised triplex-forming repeat, the (GAA)n•(TTC)n repeat (Gacy et al. Mol. Cell, 1998, 1, 583-593), is predicted to be unfolded under the condition of our assay and displays stall scores in average below 0.3 for any length and at any time point (refer to Additional File 2 | CSV file for the individual values). Hence, as expected, triplex-forming repeats do not impede DNA synthesis in single-stranded DNA.*

2. Are the high-scoring hairpins basically all the palindromic sequences (that can form a hairpin without mismatches)? Were there any other characteristics that came out of the analysis - what was learned about this class?

*Out of the 188 unique single-stranded STR motifs identified to fold into hairpin-like structure, only 37 (~ 20 %) are palindromic motifs. This observation shows that hairpin-like motifs are more diverse than expected. We have therefore performed an analysis to assess the diversity of hairpin-like STR focusing on mismatch tolerance. Using the Mfold DNA folding algorithm we predicted the most stable hairpin structures from these motifs in order to identify the bases involved in these hairpins and potential mismatches. We found, see figures below, that (i) longer STRs can accommodate more mismatches, (ii) an increased number of mismatches is associated with lower stall scores and (iii) GC base pairing (odds ratio = 1.82; Fisher’s two-sided P = 1.21 x 10-4) and A mismatches (odds ratio = 1.60; Fisher’s two-sided P = 1.42 x 10-3) are enriched in hairpin-like STRs. The last point suggests that A may stabilise mismatches and/or are involved in non Watson-Crick base interactions. As an example, we found that the CAG hairpin (A-A mismatch) induce greater DNA polymerase stalling that its relative the CTG hairpin (T-T mismatch). We have now included the following figures in the revised version of our manuscript (Additional file 1: Figure S4g-i) and discussed these findings in the main manuscript (P11 L27 - P12 L13).*

*Characterisation of STR motifs predicted to fold into hairpin-like structures.*

3. How did you determine the ideal number of nucleotide lengths to test?

*The lengths of the repeats to be tested were chosen to reflect the distribution of STRs in the human genome (see Figure below). The distribution of STR length highlights discrete enrichment of STR length at ~ 24, 48 and 72 nucleotides. Additionally, STR length that can be studied in our high-throughput primer extension assay is limited as we rely on Illumina 150 bp paired-end sequencing runs to identify STR sequences within the DNA library and 50 to 75 non-repeated DNA sequences are needed for aligning and identifying each sequence robustly. Hence the length of STR that can be studied is limited to ~ 75 nt.*

*STR length distribution in the human genome (hg19)*

Along that line, one part of this study that is largely ignored is how many bases participate in the repeat which may impact the size of the overall structure/barrier (for example, for the 72nt example, 1 nt repeat would equal 72 repeat units, 2nt=36 repeats, etc). If anything, if your longer STRs have produce a stronger stall it's more impressive because the overall number of structures that can form are fewer. Similarly, shorter STRs have more base pairing opportunities/ perhaps not all repeats are part of the structure so perhaps it's not so surprising that your polymerase travels more internal initially before stalling. In your analysis, did you uncover any relationship between STR unit length and stalling?

*We now provide an analysis on the effect of STR periodicity and of the number of STR motifs on the ability of STR to stall DNA polymerase progression (see Figures below). We found (i) an anticorrelation between STR periodicities and (ii) a strong correlation between the number of STR motifs and the stall scores. Taken together these observations suggest that shorter STR motifs have, as suggested by this reviewer, more base pairing opportunities. We have now included the following figures in the revised version of our manuscript (Additional file 1: Figure S4c-d) and discussed these findings in the main manuscript (P10 L30 - P11 L5).*

*Correlation between STR periodicity and number of STR motifs and stall scores. The graph reporting the variation of the stall score versus the number of STR motifs have been plotted using data from 24, 48 and 72 nt long STRs.*

4. Figure 2F. Your statistics are all relative to the repeat sequences and not the control. For example, are i-motif's different from the control? In p8 ln7-11 you infer that this mathematical calculation will allow you to separate out different sequences but if one of them isn't statistically different from the control, is this really true? Is the library different from the control? It looks like the median is like the control and the outlying hairpin and G4 sequences are what drive differences.

*Fig. 2f reports the distributions of the kinetic constants λ associated with DNA synthesis at the control and STR sequences. This graph highlights that each structural class (hairpins, i-motifs and quadruplexes) arising from the positive control sequences display statistically different λ values and support the idea that the λ feature can be used to differentiate each structure. We then demonstrate (Additional file 1: Figure S3) that both the strength of polymerase stalling and the kinetics of DNA synthesis (λ) are needed for predicting structures. Hence the λ feature alone allows structure classification but is not sufficiently powerful to predict structure. We do not claim that the λ values for the positive controls (hairpins, i-motifs and quadruplexes) are statistically different from those of the negative controls (random sequences of varying GC content) nor the STRs, hence these statistics are not needed. To clarify this point we have rewritten this section in the revised version of the manuscript (P8 L32 – P9 L8).*

5. P8 ln38-42. You do not show the control data or statistics to illustrate that UNF sequences are not different from control sequences. From what I can determine, you are calling these unfolded sequences because they aren't different from the control in your assay. Is this correct? These are still STR sequences, so is it possible that they are structured but missed by the constraints of your assay? I presume your control sequences aren't predicted to form secondary structures (are truly random). Are the UNF sequences predicted to form structures? It doesn't seem appropriate to utilize the UNF sequences for comparison statistics when comparing UNF, HAIRP, IMOT, and QUAD like in figure 3b, 3c, 3d, and 3e.

*UNF sequences comprises sequences whose stall scores are not statistically different from the stall scores of the negative control sequences, i.e. random sequences of varying GC content, at each time point. The exact computation is reported in the Methods section “STR structure classification using a supervised machine learning approach”. By definition, their stall scores are not statistically different from the negative control data (Q-values > 0.1). We named them UNF, for unfolded, rather than unstructured since we cannot rule out they will not be structured under different experimental conditions than those used in our polymerase extension assay. However, it worth noting that even if they could form structures, the resulting structures are not stable enough to impede polymerase progression. We added a comment in the manuscript to explain this point (P9 L25-26).*

*Polymerase stalling at STRs can be due to either their repetitive nature or their ability to fold into secondary structures. In order to assess the role of DNA structures, it is crucial to compare repeats to each other. This is why we compared the HAIRP, IMOT and QUAD to the UNF STRs rather than the negative control sequences, which are not repetitive, in Fig. 3b-e. Nonetheless because the stall scores of UNF STRs are by definition not statistically different from the negative control sequences, the conclusion will be the same.*

6. It is not clear what the sequences of your G4 and hairpin motif's are in all of your experiments. Are all your control sequences what are listed in figure legend 2A and 2B? They aren't tandem repeats—the G4 sequence is 32 nucleotides, which isn't a length you test in your library. Additionally, it seems to have only (4) G4 sequences - a tandem repeat would have 8. The hairpin sequence would also form only 1 large hairpin - an STR could form many smaller hairpins which may present differently in your assay. Using these as a control which you compare things to may skew your results. Please clarify in text what the control sequences are, why they are ideal controls for this study and what, if any, are the limitations of these controls.

*We do not fully understand this point raised by this reviewer but we try to provide here a detailed explanation on the nature of the controls used in our study.*

*The exact sequences of the 960 hairpins, 1,500 G4s, 472 i-motifs and 1,000 random sequences used as positive and negative controls are reported in the Additional File 2 as stated in the main text of the manuscript and in the Method section. Positive control sequences were designed in order to favour the formation of DNA structures of different stabilities in order to assess how DNA structures affect DNA synthesis. These sequences are not repeats and their lengths range from 12 to 48 nt (see Figure below). They are separate from the STR sequences in the library. These sequences were incorporated into the DNA library in order to assess how well-defined DNA secondary structures, i.e. non-repeat sequences, impede DNA polymerase progression. We feel that the manuscript describes the nature of the control sequences appropriately and that the Method section explains clearly how they were designed.*

*Distribution of positive control lengths*

7. Need to qualify and discuss some conclusions as you have only measured one aspect of STR replication, namely how structures in the template strand affect polymerase progression. There are many others not measured here. For example, the bias to contractions when replicating hairpins is not surprising, as it has been long known that they occur by structure formation on the template strand. In contrast, expansions usually occur by structure formation on a displaced non-template strand, something not present in this system.

*STR expansion and contraction events occur following polymerase slippage at repeated sequences and may be affected by structure formation within the non-template or template strand respectively. In the course of our project we found that unfolded STR are more prone to expansion/contraction that the structured STRs, but we also found that structured STR affect the expansion/contraction patterns. We found that while the most frequent events are expansions by a single unit, the HAIRP STRs displays more complex events. Additional File 1 Figure S8b reports a summary of all observed events and shows that HAIRP STR are more likely to expand or contract by multiple units at once (up to 4 units at a time). We observed both expansion and contraction events supporting that our assay monitors the formation of structure within the template strand to be monitored but also within the nascent (non-template) strand following DNA polymerase dissociation.*

Minor comments (items to address):

8. P4, Ln 38-42. Some evolutionary context may be helpful here. You're using a library approach initially that then ties into human genomes more generally. It would be helpful to understand how T7 DNA polymerase models Eukaryotic/mammalian replication.

*Please refer to the discussion at the beginning of the answers to the reviewers.*

9. Figure 1b and pg 6 ln15-20. What are these transient replication stalls? Could you label them in your figure? To show these are replication stalls and not just non-specific bands could you show a t=0/pre-incubation with T7 lane?

*We have now labelled the transient replication stalls on Fig. 1b. These are transient replication stalls since they disappear over time and are probably due to sequences within the template (but outside the DNA library insert) that are difficult to replicate. Showing a t=0/pre-incubation will not be informative as the reported gel are detecting the elongated fluorescently labelled primer.*

10. Figure 2a. What is represented in each bar? What is the Y-axis?

*Fig. 2a-c are density plots which represent the distribution of the stall scores of the G4, hairpin and STR sequences. The height of each bar represents the normalised number of observations per bin of stall scores. In these figures we divided the stall scores in 100 bins meaning that each bar covers ranges of 0.01 units of the stall scores. The line on top of the bars is a Kernel density estimation of the distribution of the stall scores. Because these figures are composite figures and report only qualitative information, the value of both the density and the counts are not informative and have been omitted.*

11. Figure 2c. You don't really discuss figure 2c in the text other than you ran the library against G4 and hairpin controls—What does the data mean? The median stall score? Does it change? What do you make of the "significant?" population that has a 100% stall rate for all timepoints?

*Fig. 2c is reported to allow the reader to qualitatively estimate the distribution of the stall scores linked to STRs at the different time points. The variation of the stall scores associated to the STRs over time is, as expected, more complex (wider distribution and absence of a specific pattern) that the behaviour of the control G4s and hairpins, as being much more diverse. This also suggests, as demonstrated latter in the manuscript, that STRs cover different type of structures. The population with scores of 1 results from STRs that are detected only in the stalled and not the extended fraction. These sequences have been enriched from the parental library in our primer extension assay pipeline and are greatly impeding DNA synthesis. We have now added a comment in the manuscript to clarify this point (P7 L22-26).*

12. Pg 7, Fig 2. Lambda () is not well defined. Please add a more complete description of what this parameter represents and how it was calculated.

*We have now a more complete definition of the parameter (P8, L10-17).*

13. I suggest you remove HAIRP, QUAD, IMOT (and UNF) abbreviations in the text and just call them hairpins, quadruplexes and i-motifs for better readability.

*In the manuscript, hairpins, quadruplexes and i-motifs refer to the control sequences designed to fold into these structures while HAIRP, QUAD, IMOT refer to the STRs predicted to fold into these structures. Using the same terminology would be confusing for the reader. We would therefore prefer to retain the distinction between sequences that do form these structures and those that we predict form these structures.*

14. Fig. 3a. Why was sigma at 0.5 min chosen (x axis, explain logic)? What is the Q value on the Y axis and how is that related to the stall score? This graph and how to read it should be explained better to the reader.

*Fig 3a reports the distribution of the averaged stall scores for the 964 unique single stranded STR motifs when 48 nt long together with the statistical significance of their scores. This plot, similar to a Volcano plot, enables the quick visual identification of STR motifs with high stall scores that are also statistically significant. From our data, we can generate 15 similar plots (3 STR lengths at 5 different time points) but we selected this condition as it allows the visualisation of both the repeats inducing transient and persistent stalling events. Additional file 1: Figure S4a and S4b report a similar plot for different STR lengths. As reported in the figure legend, the Q values are computed by combining the P values associated with the statistical differences in stall scores at all time points observed for a given STR motif and the negative control sequences of varying GC content. They reflect that DNA polymerase stalling at STRs is structure, rather than sequence, dependent. The exact computation methods are reported in the “STR structure classification using a supervised machine learning approach” paragraph of the Methods section.*

15. Pg 12 lines 52-55 should read "Taken together these observations suggest that STR mutational profile is intimately linked to polymerase stalling and to STR length and sequence stability."

*This sentence may have been ambiguous and we have rewritten it (P15, L4-6): “Taken together these observations suggest that STR length and sequence instability is intimately linked to polymerase stalling due to STR structure formation.”*

Reviewer #2:

The human genome contains over 4 million Short Tandem Repeats (STRs). While expansion of several STRs have been linked to pathological consequences, how STRs are constrained is poorly understood. In this manuscript by Murat et al., the authors investigate how STRs affect DNA synthesis to determine if this can explain their instability. For this, they generated a ssDNA plasmid library that contains about 20 thousand STRs covering all permutations of 1-6 nt repeats with three defined lengths. By replicating the STRs with T7 DNA polymerase, they assigned each STR into four classes (unstructured, hairpin, i-motif, and G4) based on how their replication kinetics compares to STRs known to fold into these four classes. They then sequenced the replication products and find that structured STRs are more prone to point mutations, while unstructured STRs tend to induce polymerase slippage leading to expansions/contractions. Interestingly, they also find that structured STRs are significantly shorter and less abundant compared to unstructured STRs in eukaryotic genomes including the human genome. This is likely driven by the higher degree of point mutations in structured STRs.

This is a well-executed study that provides important information on the evolution and maintenance of repeats in our genome. It is somewhat surprising that the simplified system used in this study (primer extension on ssDNA templates by a T7 polymerase) can generate information relevant to the in vivo situation. However, the data is consistent with several aspects of repetitive sequences in our genome and this system can therefore have predictive value. Although this is a new and powerful approach and fits well with this journal, there are some issues that need to be addressed before I would recommend final acceptance.

Major points:

1. How can the authors exclude that the PCR amplification of the stalled and extended products introduces PCR bias. The stalled products can be a lot smaller compared to the extended products and 35 cycles of PCR could favor amplification of these smaller products which could have a big effect on the stalling factor. Is there any evidence that this does not occur? this should at least be addressed.

*To prepare the products of the primer extension reactions for sequencing, the stalled and extended products were excised from the gel matrix and processed separately. Hence during the PCR steps, the smaller (230 nt, stalled products) and the longer species (3098, extended products) were not amplified together and no biases are expected during this step. Prior to amplification, the stalled products were tailed using Terminal deoxynucleotidyl transferase (TdT) as the primer downstream of the repeats is missing due to the stall of the polymerase. PCR amplification of these tailed products required 35 cycles as the TdT tailing reaction and subsequent purification steps recovered only a limited quantity of tailed products. Nevertheless, at this stage no GC-rich sequences nor structure-forming sequences are present in the template since the polymerase stalls upstream of the repeats (or structure-forming sequences) and no PCR biases are expected.*

*The only concern with PCR biases arises when amplifying either the parental library, i.e. the library that has not been extended by the T7 DNA polymerase, or the extended products. To minimise these biases, we optimised the PCR conditions used in our pipeline. Briefly, we screened polymerases known to efficiently amplify GC-rich templates (Roche Kapa Hifi, NEB Phusion, Agilent Herculase II etc.) and PCR conditions (denaturant, temperature, number of cycles...) to minimise biases between different repeats. Below are examples of gels comparing the Phusion and Kapa Hifi polymerases on different repeats and structured templates. We then selected the Kapa Hifi polymerase and the PCR conditions reported in the Methods section as the best conditions for maintaining a low level of bias. It is noteworthy that we used only 10 cycles of amplification. Using these conditions, we demonstrated that sequencing of the amplicons generated with the parental library introduces very little bias due to GC content (Additional file 1: Figure S1b) and is highly reproducible between repeats (Additional file 1: Figure S1d).*

*Taken together these observations support that PCR biases have been kept to a minimum and should not significantly interfere with the values of the stall scores.*

*Gel electrophoresis of amplicons generated using optimised PCR conditions and the Roche Kapa Hifi or NEB Phusion polymerases. These gels show that the performance Kapa Hifi is less baised than the Phusion polymerase.*

2. While the data generated by primer extension with the engineered T7 DNA polymerase (Sequenase) correlates with several aspects of STRs in our genome, this polymerase differs in important aspects from the mammalian replicative polymerases: it lacks exonuclease activity and it is an A-family polymerase and not a B-family polymerase. The results would gain a lot of strength if a similar assay would be performed with a B-family polymerase, at least on a selection of STRs, and similar data would be obtained.

*Please refer to the beginning of the rebuttal letter for a thorough discussion explaining our choice of the T7 DNA polymerase.*

3. In the STR library, inter-plasmid (STR) annealing might occur during heating and cooling down of the plasmids, raising the possibility of incorrect stalling scores. Is it possible to take into account the inter-STR annealing for STR classification, ie by eliminating complimentary sequences from structured STRs etc?? This possibility needs to be discussed at least.

*Polymerase extension assays were performed using 3098 bp single-stranded DNA templates at a concentration of 100 ng/μL (which correspond to a molar concentration of ~ 100 μM). Assuming that each sequence is uniformly represented within the library, each of the 16,068 STR sequence is then found at a concentration of ~ 5 pM. Such low concentration favours intramolecular over intermolecular structures. Hence inter-plasmid annealing should not affect the computation of the stall scores. If inter-STR annealing was happening, one would expect that stall scores associated with a STR sequence and its reverse complement should be similar. We have tested this hypothesis by plotting and testing the correlation between the stall score of each of the 48 nt long STR sequences against their reverse complement (see Figure below). This graph does not display such a correlation (Rho = 0.191) and supports that inter-plasmid annealing does not contribute to polymerase stalling in our high-throughput primer extension assay.*

*Graph reporting the stall scores of each single-stranded STR motif and reverse complement*

4. Both the positive control STRs that are known to fall into one of the four structural classes, and the STRs predicted to fold into these structures are referred to as ' unstructured, hairpin, i-motif, and G4'. As for most of the 'random' STRs, as it has not been experimentally shown that they fold into these structures (this is only based on the parameters determined by the data), it would be more correct to refer to these as I-motifpred, G4pred etc. (or something along these lines). This would also make this manuscript easier to follow (e.g. it would show that the data used for Fig 2a/6 and 3 is different).

*In the manuscript, hairpins, quadruplexes and i-motifs refer to the control sequences designed to fold into these structures while HAIRP, QUAD, IMOT refer to the STRs predicted to fold into these structures. We then use capital letters to distinguish between designed and predicted structures. We added a comment in the manuscript to reflect that UNF, HAIRP, IMOT and QUAD description when used to describe STR are referring to classifications/predictions (P10 L3-5).*

Minor points:

5. In two places (p9 and p16), the condition of the assay is referred to as 'physiological condition'. However, the condition greatly from a cellular context, this should be rephrased.

*The conditions of our primer extension assays (40 mM Tris.HCl pH 7.5, 20 mM MgCl2, 50 mM NaCl and 50 mM KCl) are indeed not physiological. In the two sentences highlighted by this referee, we wanted to emphasise that some C-rich STRs were able to fold into i-motifs at neutral pH. This is now clarified or removed (P10 L13; P19 L27).*

6. Figure 1b: What is the fully extended product? It is not clear which band the arrow points at. If the G4 fully stalls replication, why is there a band at the height of the extended band? Or is this not the fully extended product? In addition, how are the stalled bands isolated. For the G4, this is a single band but this experiment is performed in pools to the stalled band is likely a smear. This should be explained.

*We report below fully annotated pictures of the gels that are reported in Fig. 1b (together with the second replicate for the STR library). We labelled on these gels the transient pauses, stalled products and extended products. We noticed that the arrow pointing to the different replication products in Fig. 1b moved during the preparation of the figure, this is now corrected.*

*Concerning the G4 containing template, one can see that the majority of the primer extension reaction runs as stalled products. In fact, when quantifying the intensity of the signal, the bands corresponding to the stalled products represent 70 % of the total signal. Hence the G4 is significantly but not fully stalling DNA synthesis. We have clarified this point in the manuscript (P6, L19-22). The band corresponding to the stalled and extended products on the gels analysing the DNA library where excised using a scalpel and crushed for DNA purification and sequencing. As noted by this referee, stalled bands for the DNA library are expected to run as a smear and we cut the gel from the bottom of the band and extended it to the first noticeable transient pause sites. This information has been added in the Methods section in the “Sample preparation for sequencing” section.*

7. Although the stall score seems to be a powerful parameter, it is very difficult to understand exactly how it is calculated. In the method section the following is writing: 'Stall scores were then computed as the ratio of the number of reads associated with a sequence in the stall fraction over the total number of reads associated to the same sequence in both the stalled and extended fractions'. How is this made relative to all other sequences in the library (mentioned on page 7)? How can this score go up for the G4 structures. This needs to be explained better.

*Stall scores were computed for each sequence by dividing the number of reads in the stalled fraction by the total number of reads (stalled and extended fractions). This computation was performed using the normalised number of reads to take into account the variation of the counts for each sequence within its sequencing libraries. Hence the stall scores are relative rather than absolute values. In other words, the stall score associated with a sequence for a given experiment depends on the stall scores associated with all the other sequences. If the stall score associated with a sequence varies over time, all the other stall scores will also change because the total number of reads that has been normalised remains constant. Thus, if the stall score associated with a given sequence decreases overtime, the stall score of another sequence must increase. This point has been clarified in the manuscript (P7 L28-31).*

8. Figure 2d: The example for the G4 structure is very different from the average based on 2a, this makes 2d difficult to understand. Could a more representative example be taken?

*The example reported in Fig. 2d highlights the G4 sequence #19213 that displays stall scores varying from ~ 0.65 to ~ 0.9 over time. These values are actually very representative of the distributions reported in Fig. 2a. which display medians at 0.48 and 0.83; and maxima at 0.59 and 0.93 at 30 s and 30 min respectively (as reported in the main text). We have reorganised Fig. 2d to make this point clearer.*

9. Figure 2f: Why does the i-motif structures display positive lambda values, while they are likely to be resolved like hairpin (Figure S2a and b). This should be explained in the text. Related to this, figure S2g-I. If I understand correctly, lower lambda value means quicker synthesis after stalling. Why do hairpins with higher Tm show lower lambda values in panel g? Moreover, why do G4 and imotif structures show predominantly negative lambda values but it is concluded that these structures have positive lambda?

*λ values associated with the hairpins, i-motifs and G4s, reported in Fig. 2f, are for sequences that are structured under the conditions of our assay, i.e. that have a stall score statistically higher than the random control sequences at each time point. This information was reported in the core of the manuscript but missing in the figure legend, this is now corrected. Hence these sequences represent only a subset of all the sequences reported on Fig. 2a,b and Fig. S2a and were selected to assess the contribution of DNA structures on variation of the stall scores over time.*

*We plotted the correlations between the predicted stability of the DNA structures and their associated λ values (Fig. S2g-i) to demonstrate that the variation of the stall scores over time depends on the stability of the structure encountered by the DNA polymerase. Note that the λ parameter does not describe the rate of DNA synthesis but rather the rate of the resolution of the stalled polymerase. Hence negative and positive values are associated with transient and persistent stalling events. The observation that the λ constants correlate with the predicted stabilities of the structures (Figure S2g-i) demonstrate that structure formation, rather than the DNA sequence, affects the kinetic of stalls resolution. We have rewritten the paragraph describing these results in order to clarify this point (P8 L32 – P9 L8).*

10. Figure 3a: Is there any correlation with known pathological STRs and the authors' structure assignment or stalling scores. This would be interesting to mention.

*We report below the stall scores and predicted structure associated with pathological STRs together with the statistical relevance of the differences between their scores to the scores derived from unfolded repeats. Nevertheless, it is difficult to make firm conclusions as to the relevance of our data in the context of pathological repeats because it is likely that their expansion requires transcription (see Nakamori et al. Nat. Genetics 2020, 52, 146-159 for example). We therefore do not feel it is appropriate to report this figure in our manuscript, as we know that other mechanisms contribute to the pathological expansion of STRs.*

11. Figure 4d: The color scheme is confusing, blue/pink is also used for hairpin/G4 structure in the rest of the figure.

*The colours on Fig. 4d have now been changed.*

12. p13: 'suggesting that STRs capable of significant secondary structure are deleterious. Indeed, structured STRs are less abundant that their unstructured counterparts'. Yes, but G4s are more abundant than hairpins even though they are more structure. Therefore, this correlation does not fit exactly. How is this explained? See also in figure S13.

*Additional File 1 Fig. S13 reports the abundance of STR motifs according to their structural class in different eukaryotic genomes. These figures show (i) a negative correlation between STR abundance and stall score and (ii) a depletion of structured STR within eukaryotic genomes. We then concluded that structured STRs are deleterious to eukaryotic genomes. When analysing the impact of predicted DNA structures, one can note that hairpin-like STRs are less abundant than tetrahelical STRs. It is noteworthy that all structures lead to similar strengths of polymerase stalling in our assay after 30 s (see Fig. 3b, the comment that G4s “are more structure” is then not accurate) and different strengths after 30 min with lower score for the hairpin-like STRs (see Fig. 3c). We then concluded that even transient DNA polymerase stalling at STR loci poses a challenge to replication that may explain the deleterious effect of structured STRs. It is noteworthy that the abundance of STRs of each structural class differs and this observation may be explained by (i) how transient and persistent replication stalls are resolved, (ii) the existence of structure specific binding proteins and helicases, (iii) positive or negative selection processes at structured STRs or (iv) the prevalence of replication-independent mechanisms that contribute to STR length variation and abundance.*

13. Related to this, figure 4e: The authors argue that structured motifs are enriched in the pool of STRs exhibiting point mutation, but actually only G4 structures appear to be enriched as shown in Figure S9c. To make this point clear, it is better to compare the point mutation frequency among UNF and other respective structures like Figure S8a. Also Figure S8a should have UNF to directly compare slippage frequency between unstructured STR and structured species.

*We now report the frequencies of expansion/contraction and base substitution events associated with the UNF STRs together with the statistical analyses comparing the structured and UNF STRs (see Figures below). These plots show that structured STRs are less and more prone to expansion/contraction and base substitution than the UNF STRs respectively. Additional File 1 Fig. S8a and S9c have then been updated.*

14. Figure 5f: The authors state that "the higher the stall score of a STR, the higher the density of SNPs at the boundary of the locus", but actually is there significant difference between medium and high sigma values? This point should be clarified.

*There is indeed a significant difference between SNP densities at position surrounding high stall score and medium stall score STRs (Fisher’s two-sided < 2.2 × 10−16). This point has been clarified in the manuscript (P18, L12-14).*

15. Several supplementary Figures are not referred to in the main text.

*This is now corrected.*

Reviewer #3:

This is an elegantly simple and powerful study of a DNA polymerase stalling behavior of all possible STR with very clear results and conclusions supported by adequate statistical analysis. The limits of the study are nevertheless clearly stated and discussed, mostly in the discussion. This is one of the rare times where I have no serious criticism or pressing question about a manuscript.

Still, it is not clear to me to which point the behavior of T7 DNA polymerase can be taken to reflect the behavior of the replicative DNA polymerases in the organisms studied, whether in terms of stalling, slippage or error-prone DNA synthesis. The implicit reasoning of the authors is that such events in their deliberately simple in vitro system are sufficiently similar to the in vivo DNA replication process to explain the observed size and abundance distributions and SNP densities around simple repeats. This is probably true, given the striking correlations observed, but some mechanistical justification based on existing literature would be welcome if there is any beyond the authors' observations.

This work is rather unique and potentially very useful to researchers in the field. I would have no objection to accept this manuscript especially if they can answer my single concern above.

*Although the structure and organisation of the different domains of the T7 DNA polymerase (A family) and the eukaryotic replicative polymerases (B family) differs, the polymerase catalytic cores share significant structural similarities (Johansson et al. Cold Spring Harb. Perspect. Biol. 2013, 5, a012799) that may explain why the data from our in vitro assay reflect the behaviour of STRs in eukaryotic genomes. We feel that the domains that contribute the most to how polymerases behave at DNA structures are the catalytic site and the “channel”, i.e. the cleft where the single-stranded DNA binds. Indeed, hampered accessibility to the active site and/or a decrease affinity to the channel may explain how DNA structures induce polymerase stalling and/or dissociation. These two domains are actually the most conserved domains between polymerases of the A, B and X families (see Wu et al. Chem. Rev. 2014, 114, 2759−2774 for a complete analysis), which may explain why the behaviour of the T7 DNA polymerase at DNA structures in vitro correlates so well with the behaviour of STRs within eukaryotic genomes. We appreciate that more information about the molecular interactions between polymerases and STRs and DNA structures will be useful to understand this point further.*

*Please, also refer to the discussion at the beginning of the answers to the reviewers for a thorough discussion on the use of the T7 DNA polymerase in our high throughput primer extension assay.*

**Second round of review**

**Reviewer 1**

The authors have addressed all of our concerns. This is now a much better written study and I like the points they added. It should be accepted from my viewpoint.

**Reviewer 2**

The revised version of this manuscript contains several additional computational analysis and the authors provided a very elaborate rebuttal to address the comments of all reviewers. My main issues were all properly addressed. Although I would still be very interested to see if this approach using a B-form polymerase would yield a similar result, I understand the rationale provided for not doing this and agree that it likely to have a comparable outcome. I appreciate all the clarifications that have been added to the manuscript and recommend publication without further delay.
